# Comparing likelihood-based and likelihood-free approaches to fitting and comparing models of intertemporal choice

**DOI:** 10.3758/s13428-025-02779-z

**Published:** 2025-08-11

**Authors:** Peter D. Kvam, Konstantina Sokratous, Anderson K. Fitch, Jasmin Vassileva

**Affiliations:** 1 The Ohio State University, 1835 Neil Ave, Columbus, OH 43210 USA; 2 University of Florida, Gainesville, FL USA; 3 Virginia Commonwealth University, Richmond, VA USA

**Keywords:** Amortized inference, Simulation-based inference, Delay discounting, Neural networks, Deep learning

## Abstract

Machine learning methods have recently begun to be used for fitting and comparing cognitive models, yet they have mainly focused on methods for dealing with models that lack tractable likelihoods. Evaluating how these approaches compare to traditional likelihood-based methods is critical to understanding the utility of machine learning for modeling and determining what role it might play in the development of new models and theories. In this paper, we systematically benchmark neural network approaches against likelihood-based approaches to model fitting and comparison, focusing on intertemporal choice modeling as an illustrative application. By applying each approach to intertemporal choice data from participants with substance use problems, we show that there is convergence between neural network and Bayesian methods when it comes to making inferences about latent processes and related substance use outcomes. For model comparison, however, classification networks significantly outperformed likelihood-based metrics. Next, we explored two extensions of this approach, using recurrent layers to allow them to fit data with variable stimuli and numbers of trials, and using dropout layers to allow for posterior sampling. We ultimately suggest that neural networks are better suited to fast parameter estimation and posterior sampling, applications to large data sets, and model comparison, while Bayesian MCMC methods should be preferred for flexible applications to smaller data sets featuring many conditions or experimental designs.

**Supplementary Information:**

The online version contains supplementary material available at 10.3758/s13428-025-02779-z.

## Introduction

A central goal of research in psychology is to make inferences about latent cognitive processes from observed behavior. Methods for assessing processes like risk-taking, delay discounting, learning, memory, or cognitive control can help us understand both healthy and disordered decision behavior, as well as link behavior in the laboratory to important outcomes of interest like substance use and mental health (Bickel & Marsch, 2001; Lejuez et al., 2003; MacKillop et al., 2011; Zois et al., 2014).

However, behavior alone can be unreliable (Hedge et al., 2018). This can be rectified by building stronger generative models, using computational cognitive modeling (Haines et al., 2025, 2023; Sullivan-Toole et al., 2022) and informative constraints such as those imposed by (hierarchical) Bayesian analyses (Molloy et al., 2020; Kvam et al., 2024). Cognitive models of behavior are able to capture the latent cognitive and neural processes that give rise to behavior on clinically diagnostic tasks (or even across multiple tasks; Kvam et al., 2021), thereby linking decision deficits in clinical populations to differences in mental functions (Busemeyer & Stout, 2002; Wallsten & Pleskac, 2005; Yechiam et al., 2005). These models can detect neurocognitive markers of substance use and mental health disorders even in cases where behavior by itself is uninformative (Romeu et al., 2019; Ahn et al., 2016).

Recent innovations in machine learning have made it possible to train neural networks to fit, compare, and evaluate cognitive models (Kvam et al., in press; Radev et al., 2020, 2021; Sokratous et al., 2023; Lenzi et al., 2023; Sainsbury-Dale et al., 2025, 2024; Zammit-Mangion et al., 2024). In doing so, we are able to *amortize* these computations, offloading the model creation, comparison, and fitting process from a user to a modeler who trains a supervised learning algorithm to perform parameter estimation or model comparison (Elsemüller et al., 2024). In the past, these applications have focused mainly on models that need to be simulated, as these pose the greatest challenges to developing and testing novel models (Fengler et al., 2020; Sokratous et al., 2023). However, these same approaches can also be used to fit traditional likelihood-based models. In terms of testing machine learning approaches to model fitting, such models may indeed be the most informative because the likelihood provides a benchmark against which we can compare the new approaches.

The first goal of this paper is to provide a thorough test of these new approaches to model fitting by comparing trained neural networks against more traditional (but still successful) approaches using hierarchical and non-hierarchical Bayesian modeling (Shiffrin et al., 2008; Molloy et al., 2020) in terms of both their performance and fitting time. We focus on delay discounting and its relationship to substance use (Bickel et al., 2014, 2012), where models are used as tools for both diagnosis and prognosis of individuals who are at-risk for developing a substance use disorder, undergoing treatment, susceptible to relapse, or candidates for early intervention programs (Bickel et al., 2011; Conrod et al., 2010; Donohew et al., 2000; Vassileva & Conrod, 2018). Our goal is to demonstrate the utility of this approach for analyzing delay discounting data, with an eye toward broadening access to modeling and its benefits related to reliability, predictive validity, and theoretical progress.

The second goal of the paper is to evaluate how similar network-based approaches perform for the purposes of model comparison. To evaluate which of two theories provides the best quantitative account of a set of data, most common approaches rely on likelihood-based metrics like AIC, BIC, or WAIC (Raftery, 1995; Akaike, 1998; Watanabe & Opper, 2010). However, these approaches are again limited to models for which a likelihood can be obtained, restricting the scope of theories we can consider. As with parameter estimation, model comparison can be amortized via machine learning. In this approach, model comparison is treated like a classification problem – determining the probability that a particular data set was generated by a particular model. By generating a large volume of data from different models and storing an indicator that corresponds to the generative model, we can train a classifier to determine which of a set of models has the greatest posterior probability (Radev et al., 2021). Here, we compare these classifications against the winners of model comparison metrics like AIC, BIC, and WAIC to determine how well a machine learning classifier does at model comparison.

Third, this paper incorporates and tests two recent innovations in neural networks for parameter estimation and model comparison. First, we show how the neural networks can be augmented with recurrent layers to handle data sets that vary in terms of the stimuli and number of trials (Rmus et al., 2023; Radev et al., 2021). This permits time-varying models as well as variations in experimental procedure (such as the order of trials) across participants. Our initial investigations have a fixed input structure by virtue of focusing on a task with a fixed set of stimuli and number of trials, but we show how this can be generalized to a wide range of paradigms and stimulus sets in the “Input structures and posterior sampling” section.

Second, we use dropout layers in the neural network to force it to vary its predictions from iteration to iteration (Gal & Ghahramani, 2016). This allows for neural network-based sampling, approximating a full Bayesian posterior distribution from a Gaussian process. We compare this against hierarchical and non-hierarchical Bayesian posteriors to evaluate network performance in terms of both accuracy and sampling efficiency. As before, we compare the resulting networks against more established Markov chain Monte Carlo [MCMC] approaches, this time focusing on the full posterior and on experimental designs with varying stimuli and trial numbers.

Put together, we present an introduction to these machine learning methods, apply them to a widely used task (monetary choice questionnaire), benchmark their parameter estimation performance against both hierarchical and non-hierarchical Bayesian MCMC approaches, compare their model comparison conclusions against likelihood-based model fit metrics, show how it can be used to fit and compare models of intertemporal choice on real data, extend neural networks to variable stimuli and trials, and finally show how they can be used to create a complete characterization of uncertainty in the form of a Bayesian posterior. To preview the results, machine learning generally provides comparable (but much faster) parameter estimates and posterior distributions, while classification networks substantially outperform contemporary model fit metrics on model comparison problems.

## Simulation studies comparing MCMC and neural networks

The first step of evaluating these new approaches is to see how well they perform on data sets where the true parameters are known. Here, we compare neural networks and likelihood-based metrics on a clinically diagnostic measure based on intertemporal choice. In these problems, a decision-maker is faced with choices between immediate and delayed payoffs – such as $10 today or $20 in 5 weeks. Their ability to defer rewards, referred to as delay discounting, is thought to be indicative of impulsivity and is related to a variety of clinical outcomes (Amlung et al., 2019). A person’s preferences for delayed versus immediate rewards are often quantified using a discounting rate, which quantifies how much a payoff decreases in subjective value as it is removed in time from the present (Yi et al., 2006; Kvam et al., 2023).

Traditionally, models of intertemporal choice have been fit using heuristic methods for estimating discounting rates (Madden & Johnson, 2010; Odum, 2011) including titration methods that progressively manipulate stimulus features to identify a function that fits indifference points (Mazur, 1987; Sawicki & Białek, 2016), using the area under the curve for indifference points (Myerson et al., 2001), using matching procedures for identifying indifference points directly from participant responses (Hardisty et al., 2013) or simple rules for estimating these values for a fixed set of choice pairs (Kaplan et al., 2016). The latter uses a fixed experimental paradigm, such as the monetary choice questionnaire [MCQ] (Kirby et al., 1999a, b). These fixed questionnaires have become particularly popular in clinical applications, as they are relatively straightforward to use in assessments and simple to code in terms of properties like discounting rates (Kaplan et al., 2016).

To preview the results, the simulation studies we present here show that (a) neural networks and MCMC (both hierarchical and non-hierarchical) Bayesian methods achieve similar levels of performance when estimating best-fit parameters, and (b) that a classification network achieves much better performance than likelihood-based fit metrics when comparing competing models.

We focus on these fixed questionnaires first, both because they are widely used in clinical settings and because they provide a simple data structure that we can use for machine learning. Specifically, behavior on the 27-item MCQ can be summarized with 27 bits: a 1 for every problem they chose the larger later option, and a 0 for every problem they chose the smaller sooner option. It can therefore be input as a 27-entry vector into a neural network, without any need for further summarizing the data or transforming it to a uniform input structure (Sokratous et al., 2023).

**Table 1:** Approximate time to complete each step of the process of model fitting for the hierarchical Bayesian MCMC approach, non-hierarchical MCMC approach, and simulation inversion neural network approach. Times are approximate and rounded to the nearest second; times taking less than 1 s are labeled accordingly

Approach	Simulation	Training	Fitting	Fitting	Posterior	Total	Total
	100k Ps	5000 epochs	300 Ps	3000 Ps	5000 samples	300 Ps	3000 Ps
Non-hierarchical MCMC	-	-	219s	2033s	-	219s	2033s
Hierarchical MCMC	-	-	225s	2089s	-	225s	2089s
Neural network	17s	82s	< 1s	5s	< 1s	105s	120s

### Neural networks

For the MCQ, we trained a neural network to map behavior (27 binary choices) onto the parameters of three different cognitive models. The neural networks were trained by simulating data from each model, fitting the parameters of a deep network using supervised learning, and then using the trained network to make predictions about known parameter values (validation set) and unknown parameter values (real data).

**Fig. 1:**
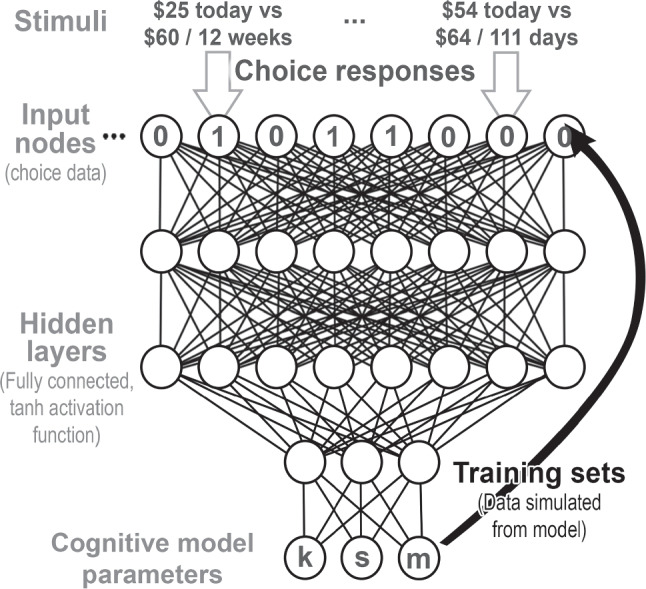
Diagram of the structure of a neural network trained to map intertemporal choice data (*top*) onto the parameters of a cognitive model (*bottom*). To train the network, simulated data (training sets) are generated from varying parameter values so that the simulated data can be used as an input to the network (*thick black line*) with the true parameter values as the desired outputs

A diagram of this approach is shown in Fig. 1. First, the parameters of a cognitive model (bottom nodes of Fig. 1) are drawn from a prior distribution specifying plausible parameter values that we might observe in a real population. The exact priors are specified for each model below, near the end of the subsections for each model (hyperbolic, hyperboloid, direct difference). Next, these parameters are used to simulate a synthetic participant – probabilistically generating responses to each of the 27 MCQ questions based on the randomly sampled parameters of the cognitive model. This process is then repeated a large number of times to create a data set of many synthetic participants and their associated model parameters. In our case, we simulated 100,000 participants from each model.

Once a synthetic data set is created, it can be used to train a machine learning algorithm what the relationship between data (27 MCQ responses) and associated model parameters (used to generate the synthetic data) should be. Effectively, what we are trying to do is use a neural network to take the simulation function $\Theta \rightarrow D$ that generates data *D* from parameters $\Theta$ and invert it, obtaining the generative parameters from the data ($D \rightarrow \Theta$. Therefore, we refer to these algorithms as *simulation inversion networks*. A separate network is trained for each of the models.

The structure of the neural networks we use for estimating the parameters of each model is based on prior work using deep neural networks for parameter estimation (Sokratous et al., 2023). The input layer consists of 27 nodes, one for each choice response on the MCQ. There are then three hidden layers of decreasing size, with 100 nodes, 66 nodes, and 33 nodes in the first, second, and third hidden layer, respectively. Each layer is fully connected to its preceding and successive layer, and the summed inputs to each node in each hidden layer are transformed using the *tanh* activation function before being passed to the next layer. The final layer of the neural network is a regression layer with *n* nodes, where *n* is the number of parameters in the cognitive model the network seeks to estimate.

The hyperparameters – like the number of layers, types of activation functions, number of nodes in each layer, regularization and learning rates – were set based on the structure of the slightly more complex models used by Sokratous et al. (2023) rather than fine-tune to the particular models and data we used here. These networks are likely somewhat larger than necessary, as the models and data are relatively simple and therefore likely to be relatively low-dimensional. Greater fine-tuning could likely reduce the training and fitting times reported in Table 1, but are likely to do relatively little in terms of improving performance, which appeared in most cases to be near ceiling.

The set of simulated data used to train the network was split into a training set of 80,000 synthetic participants and a validation set of 20,000 synthetic participants. Each of the networks was trained using the ADAM algorithm (Kingma & Ba, 2014), using a maximum of 5000 epochs, L2 regularization of 10$^-7$, and a mini batch size of 1000. All other settings followed the default MATLAB deep learning toolbox settings.

We used relatively standard loss functions for all networks. For the parameter estimation networks, the loss function was mean squared error. For model comparison or classification, the loss function was binary cross-entropy.

To evaluate how well the neural networks recovered the parameters of each model, we took the true values of the parameters from the test set and compared them against the estimates from the neural network trained to estimate those parameters. In the plots we show below, parameter recovery is quantified using the linear correlation between the true parameter values and the estimated parameter values from the test set. This allows us to avoid inflating the model performance based on fits to the training set, although in general overfitting was not an issue in any of the networks we present below.

### Intertemporal choice models

To test the performance of the neural networks trained to estimate each of the cognitive models of the MCQ, we evaluated their ability to recover individual-level parameters against that of hierarchical and non-hierarchical Bayesian algorithms that use Markov chain Monte Carlo estimation to recover the generative parameters (Shiffrin et al., 2008; Kruschke, 2014). Both the neural networks and Bayesian fitting were carried out on the test sets that the network had not seen before (in training or in validation) using 20,000 simulated participants from each model. The hierarchical Bayesian approach used uninformative priors, fit data in batches of 200 participants, used four chains of 4500 samples (1500 burn-ins) each. The non-hierarchical Bayesian approach used informative priors that matched the priors used to generate the data, mimicking the match build into the neural network. For both hierarchical and non-hierarchical MCMC methods, we present the maximum a posteriori estimates, which in practice are very close to the mean of the posterior due to the symmetry of the posterior. The code for each approach can be found on the Open Science Framework at osf.io/y4z82.

To preview the results, there were no substantial differences between the neural network and hierarchical Bayesian MCMC methods, and the non-hierarchical MCMC method was only slightly below that of the other two approaches. Otherwise, their performance in all three cases is essentially identical.

#### Hyperbolic model

The first model we examined is the classic hyperbolic discounting model (Mazur, 1987). In this model, the value of a payoff *x* decreases monotonically as it is pushed further away in time, such that the value of a payoff *x* at delay *t* is given by

1 $$\begin{aligned} v(x,t) = \frac{x}{1 + k \cdot t} \end{aligned}$$

The free parameter *k* is the *discounting rate*, controlling how quickly an option decreases in value as its delay to receipt increases. Larger values of *k* correspond to greater discounting and thus greater impulsivity – corresponding to more choices in favor of the smaller, sooner option.

While being relatively simple, this model is able to produce preference reversals – where adding a fixed delay to the smaller sooner and larger later options flips preferences from the former to the latter – while models like exponential discounting are not (Stevenson, 1986; Green & Myerson, 1996; Madden et al., 1999). The hyperbolic model, and its discounting rate *k*, are widely used to model delay discounting and behavior on intertemporal choice problems (Odum, 2011; Ainslie & Haslam, 1992; Amlung et al., 2019). To make a choice, a decision maker needs to evaluate the subjective value of two or more delayed prospects, comparing $\frac{x_1}{1 + k \cdot t_1}$ against $\frac{x_2}{1 + k \cdot t_2}$. Deterministic models focus on where a decision maker flips from selecting the smaller sooner to larger later option or vice versa. In these cases, a modeler typically fits this hyperbolic discounting function to the locations of indifference points generated from the experimental procedure as a regression.

Human behavior tends not to be deterministic, which has led hyperbolic choice models to include softmax or logistic choice rules that map differences in subjective value between two options to a choice probability (Dai et al., 2016). This step allows the model to be fit using likelihood-based methods (Myung, 2003) and makes it possible to use a variety of choice stimuli rather than titrating specific dimensions of the stimuli to try to identify indifference points. With this component included in the model, the likelihood of selecting one option over the other is proportional to the difference in subjective value between them, $d = v(x_1,t_1)-v(x_2,t_2)$.

2 $$\begin{aligned} Pr(Choose 1 > 2) = \frac{1}{1 + e^{-d \cdot m}} \end{aligned}$$

The free parameter *m* controls the variability in a participant’s choices. Large values of *m* lead to more random choice responses, while smaller values lead to more deterministic responses based on differences in subjective value between options. Note that it is entirely possible to use other choice rules, such as Luce choice (Luce, 1959) or an inverse cumulative density function like the inverse-normal CDF $\Phi$. The choice of choice rule is often thought to make little difference in the overall conclusions of a study, but it can affect the outcomes of some model comparisons (Zilker, 2022). There is no reason in principle why the approach we show here could not be applied to these different choice rules; logistic tends to be the fastest for large simulations because no approximation is needed to get a choice probability from a subjective value difference (as might be needed for a cumulative normal rule, for instance).

**Fig. 2:**
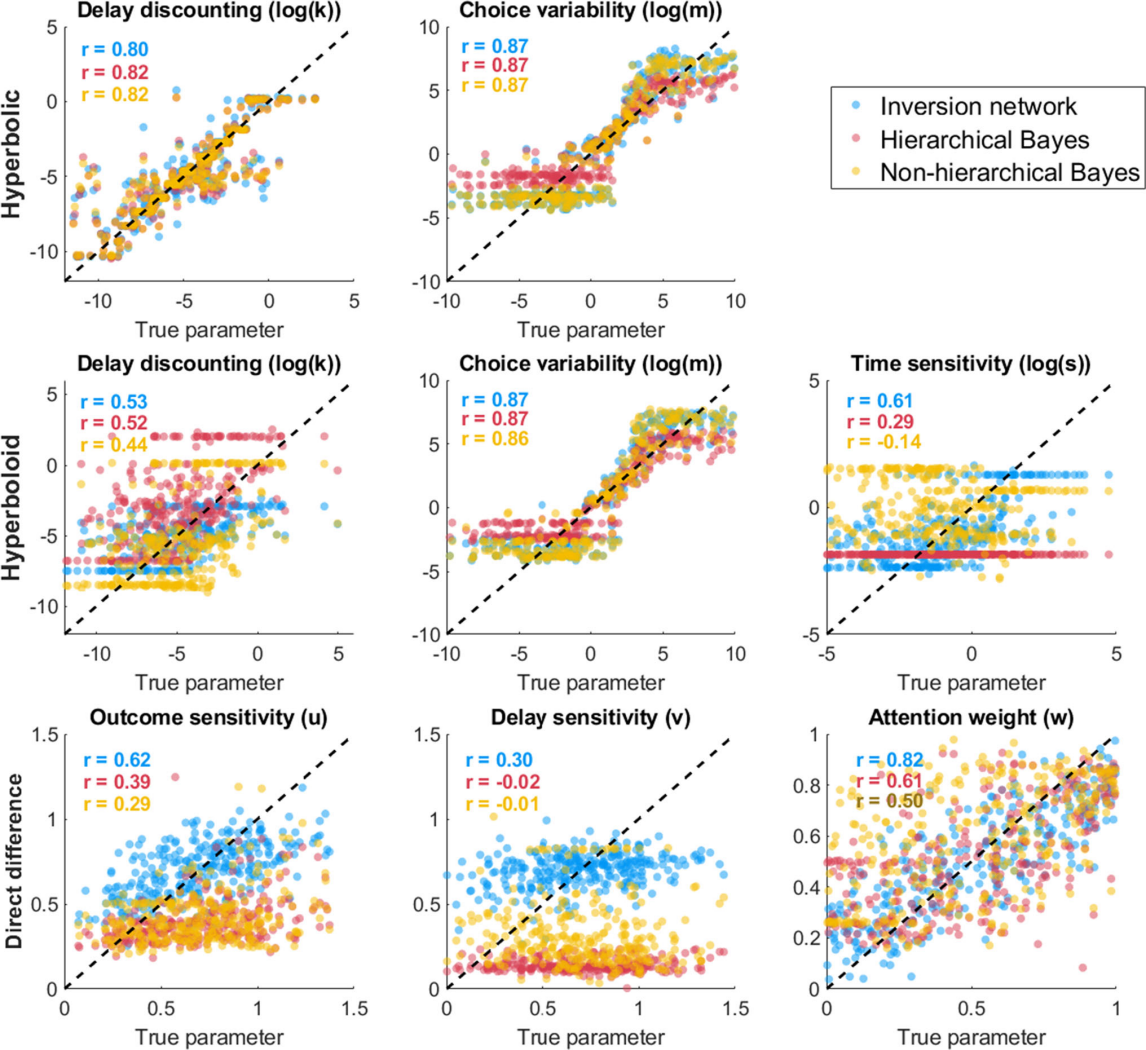
Relationship between true (*x*) and estimated (*y*) parameter values for estimates generated by the simulation inversion network (*blue*), a hierarchical Bayesian implementation of the models (*red*) and a non-hierarchical implementation of the models (*yellow*) for the hyperbolic discounting model (*top*), hyperboloid discounting model (*middle*), and direct difference model (*bottom*)

The hyperbolic discounting model was implemented in both a hierarchical Bayesian framework (Molloy et al., 2020) and the neural network approach we described above. We generated 100,000 simulated data sets from this two-parameter model, varying the log discounting rate as $\log (k) \sim N(-5,3)$ and the log choice variability as $\log (m) \sim N(1,5)$. These provided very wide ranges of both discounting rates and choice variability, aimed at ensuring that we could capture extreme behavioral data on either end of the spectrum.

For the hierarchical Bayesian model, we fit participants’ data in batches of 200, with the aim of balancing the informativeness of the hierarchical priors (group-level information shared across individuals) with information in the individual-level data. This was also done to make the computations manageable, as fitting more than 200 participants can lead to extremely long fitting times when done in a hierarchical Bayesian way. Note that this is a clear advantage of the neural network approach, which takes a matter of seconds to fit even very large data sets consisting of millions of participants (von Krause et al., 2022). It is potentially possible to identify optimized batch sizes of data for the hierarchical Bayesian approach that would identify the best trade-off between group-level and individual-level constraints; however, this is almost never done in practice (Kruschke, 2021).

The results of this network are shown in Fig. 2. Generally, both the hierarchical Bayesian and neural network approaches were able to accurately recover both parameters of the hyperbolic model with no trouble. They tended to have some trouble below and above certain values of $\log (k)$, in large part because behavior outside the range $-5< \log (k) < 1$ results in essentially deterministic behavior where a participant always chooses the SS option (if it is too high) or always chooses the larger later option (if it is too low). Overall, the performance of the neural network in parameter recovery is essentially identical to the hierarchical and non-hierarchical Bayesian MCMC estimates.

#### Hyperboloid model

The same simulation, network training, and model fitting procedures were followed for two other models, the modified Rachlin or hyperboloid model (Rachlin et al., 1986; Rachlin, 2006; Vincent & Stewart, 2020) and the direct difference model (Dai & Busemeyer, 2014; Dai et al., 2018; Cheng & González-Vallejo, 2016). These models represent some of the most widely used models in intertemporal choice as well as some of the most successful in explaining intertemporal choice phenomena (Dai & Busemeyer, 2014). We therefore expect that estimates from these models will provide complementary perspectives on behavior on intertemporal choice problems, and that comparisons between them will be insightful in terms of arbitrating between attribute-comparison or alternative-comparison theories of decision-making (Cheng & González-Vallejo, 2016; Scholten & Read, 2010; Scholten et al., 2014).

The modified Rachlin hyperboloid model adds one additional parameter over and above those included in the hyperbolic discounting model. This parameter is denoted *s*, and it quantifies how sensitive a participant is to differences in delays. In place of the subjective value given by Equation 1, the value of a delayed prospect is instead given as:

3 $$\begin{aligned} v(x,t) = \frac{1}{1 + (k \cdot t)^s} \end{aligned}$$

Note that this differs slightly from the original formulation $\frac{1}{1+kt^s}$, due to the *muddled units* problem raised by Vincent and Stewart (2020). The issue with the original model is that the units of *k* are in $(\frac{1}{days})^s$, meaning that estimates of *k* will be biased by estimates of *s*. The simple adjustment in Equation 3 addresses this issue. In this model, values of *s* close to 1 correspond to linear time perception, so that the subjective difference between 1 and 2 days is the same as the subjective difference between 101 and 102 days. Smaller values of *s* make differences among smaller values more subjectively meaningful, while larger values of *s* make differences among larger values more subjectively meaningful. The value of *s* is often taken as a subjective perception of time on a mental time line (Bonato et al., 2012).

As before, we simulated 100,000 artificial data sets of responses to the 27 MCQ questions, simulated from the modified Rachlin model. The parameters used for the simulated data were drawn randomly from prior distributions of $\log (k) \sim N(-5,3)$, $\log (m) \sim N(1,5)$, and $s \sim N(.8,.3)$ – corresponding to very loosely informative values that would be plausible for each parameter of the model. These 100,000 synthetic participants were split into training and validation sets. There were no substantial differences between performance on training and validation sets, indicating that the network was not overfitting. As before, the hierarchical Bayesian approach took these data sets and fit them in batches of 200 simulated participants, while the trained neural network fit each individual separately. The neural network was again trained on the log-transformed values of each of the parameters to ensure that its predictions for the parameter values all fell into positive values.

The results for the hyperboloid model are shown in Fig. 2. These are quite similar to the results of the hyperbolic model, and the neural network and Bayesian MCMC approaches perform almost identically. Both models had some difficulty in fitting the time sensitivity parameter *s*, although this difficulty was greater for the MCMC methods. This might be due to the stimuli in the monetary choice questionnaire having no delay for the smaller-sooner option ($t_{SS} = 0$), meaning that there was relatively little variation in delays for the model to pick up on the shape of the delay sensitivity function. Nevertheless, neither fitting approach has a clear advantage over the other and both appear to do a reasonable job of recovering the true parameter values that were used to generate the artificial data.

#### Direct difference model

The final model we tested was the direct difference model, which posits that people make intertemporal choices based on weighted differences in subjective payoffs and subjective delays. Instead of computing an overall subjective value for each option, this model suggests that people make intertemporal choices by directly comparing the attributes between their two options. It is often referred to as an *attribute-wise* model for this reason, as opposed to an *alternative-wise* comparison model.

For this model, the weighted difference *d* between the value of option 1 (with payoff $x_1$ and delay $t_1$) and the value of option 2 (with payoff $x_2$ and delay $t_2$) is given as:

4 $$\begin{aligned} d = w \cdot (x_1^u - x_2^u) - (1-w) \cdot (t_1^v - t_2^v). \end{aligned}$$

The free parameter *u* corresponds to the sensitivity to payoffs: values close to 1 indicate linear utility as a function of payoff, while values less than 1 correspond to diminishing marginal utility (and of course, values greater than 1 correspond to increasing marginal utility). The parameter *v* indexes the same type of sensitivity, but to values of time. As before, larger values correspond to increasing marginal sensitivity (greater sensitivity to larger delays relative to smaller delays) and smaller values correspond to decreasing sensitivity – such as the difference between 1 and 2 days feeling subjectively greater than the difference between 60 and 61 days. Finally, the free parameter *w* is a weight that describes the relative allocation of attention or importance to payoff versus delay. Larger values of *w* correspond to greater relative value of payoffs, whereas smaller values correspond to greater relative importance given to delays. Put together, greater values of *d* lead to a greater preference for the first option ($x_1$, $t_1$) over the latter ($x_2$).

In contrast to the hyperbolic and hyperboloid models, the direct difference model does not add any additional parameters to predict the choice probability from the difference in subjective value. Instead, it works similar to a random utility model (Dai et al., 2016) in that the variability in the attributes of the options translate into choice variability. Specifically, the variability in the difference in subjective value $\sigma$ is given as

5 $$\begin{aligned} \sigma = \sqrt{w \cdot (x_1^u - x_2^u)^2 + (1-w) \cdot (t_1^v - t_2^v)^2 - d^2}. \end{aligned}$$

The values for each of the free parameters, payoffs, and delays are the same as above. The utility difference between the smaller sooner and larger later options then follows a normal distribution $N(d,\sigma )$. Therefore, the probability of selecting option 1 over option 2 is given as the cumulative density of a normal distribution from $-\infty$ to *d*, rescaled as $\Phi (\frac{d}{\sigma })$.

With this specification complete, a modeler can sample *u*, *v*, and *w* parameters from the direct difference model to generate simulated data sets on the MCQ and estimate them using our neural network fitting approach or a hierarchical Bayesian method. These were carried out in the same way as the hyperbolic and hyperboloid (modified Rachlin) models. The priors for the three parameters were $u \sim N(.8,.3)$, $v \sim N(.8,.3)$, and $w \sim U(0,1)$, where *N* is a normal distribution and *U* is a uniform distribution. As with the hyperbolic and hyperboloid models, these values were chosen to be loosely informative, providing minimal information about what parameter values were plausible a priori.

The results are shown in Fig. 2. All three approaches recover the attention weight parameter *w* well, but vary in terms of how well they recover the outcome sensitivity parameter *u* and delay sensitivity *v*, with the neural network providing a marginal improvement over the MCMC fitting approaches.

Put together, the differences among fitting methods slightly favor the neural networks over either hierarchical or non-hierarchical Bayesian MCMC methods. It is possible that different samplers might approach the performance of the simulation inversion network more closely, but it is clear that the network is fitting as well or better than relatively standard methods for MCMC estimation (Molloy et al., 2020; Haines et al., 2025).

### Efficiency

In addition to comparing neural network and MCMC sampling approaches in terms of their accuracy, we can also compare the approaches in terms of how quickly they reach their conclusions. Especially in the case of parameter estimation, where there is little daylight between neural network and MCMC approaches, efficiency may be a deciding factor in what approach a modeler chooses.

The neural network introduces several additional steps to the fitting process beyond MCMC sampling. Before fitting, a modeler needs to create a (simulated) training, validation, and test set, and train the neural network. After obtaining maximum a posteriori estimates (either simultaneously or separately), posterior samples are then drawn by passing the same data into the network multiple times. Each one of these can add time, but the question is whether the time they add balances out the time the network can save during estimation.

To investigate this question, we recorded and averaged the time it took to simulate, train, fit, and sample using each approach. These were carried out using MATLAB 2024b on a desktop computer with an Intel i9-13900KF (3.0–5.7 GHz) processor and an NVIDIA 4070 RTX GPU. Where possible, the MCMC chains were parallelized and the neural network computations used the GPU.

It is critical to note that this analysis is not meant to be a comprehensive study investigating the exact timing of each component, but an illustrative example of roughly how long it takes to carry out each step of the model fitting process using either approach. The actual time it takes to create the training set can differ quite substantially based on how well the simulator is optimized (MATLAB’s just-in-time compilation offers a potential advantage over other methods) as well as the degree to which a modeler wishes to optimize the hyperparameters of the neural network (Zammit-Mangion et al., 2024; Elsemüller et al., 2023; Kapoor et al., 2024). Likewise, the simplicity of the input data facilitates relatively fast training, without the need for huge numbers of parameters, recurrence, or other computationally demanding pieces in the network. Still, we believe some investigation of the timings that it took for this particular application will give readers an impression of the amount of time they could expect modeling to take with the neural network relative to MCMC methods.

The results are shown in Table 1. As it illustrates, the training time is the biggest hurdle for the neural network, with other processes (even simulation) taking an almost negligible amount of time. However, even with this front-end investment, the neural network is considerably faster than either the hierarchical or non-hierarchical MCMC sampling approaches, even when the latter is optimized and parallelized.

The difference between the two approaches grows considerably as the number of participants increases. The main process that increases as more participants are added is the fitting/posterior sampling process, which takes several orders of magnitude longer with traditional MCMC approaches than it does for the neural network. As a result, the fitting time goes from $\sim 2$ times longer for a data set of 300 participants to nearly 20 times longer for a data set of 3000 participants. This difference will naturally grow as sample sizes increase. As a result, the neural network is a viable approach for handling big data where traditional MCMC sampling is not (von Krause et al., 2022).

**Table 2:** Confusion matrix for neural network-based approach to model comparison, based on simulated data from the MCQ

		Inferred model		
		Direct difference	Hyperbolic	Hyperboloid
True model	Direct difference	72.47%	5.37%	22.16%
	Hyperbolic	16.09%	51.09%	32.82%
	Hyperboloid	12.27%	1.66%	86.07%
	Total	100.83%	58.12%	141.05%

As we noted above, it is critical to note that this is not an exhaustive comparison between approaches and is meant mainly to be illustrative in terms of the potential performance of the two methods. It is worth noting that the neural network was not optimized in terms of hyperparameters, meaning that training was likely slower than it could be. Likewise, another MCMC sampling algorithm like Hamiltonian Monte Carlo (Neal et al., 2011), and different programs like Stan or PyMC (which we also examined to benchmark against the performance of JAGS) might yield better results. Despite these limitations, we can highlight a few main results. First, the neural network is in the same ballpark as MCMC approaches at worst and several orders of magnitude faster at best. Second, most of its fitting time comes from training the neural network, which only has to be done once. This time can be cut substantially by using smaller training sets,^1^ by using better hyperparameters than we did (reducing the number of nodes or layers is viable, for example), and by stopping training early when performance asymptotes (we used 5000 epochs regardless of changes in training or validation performance). Finally, MCMC approaches for even very simple models like those used in intertemporal choice become burdensome when dealing with big data in a way that neural networks do not. Because training is the main obstacle rather than fitting, the latter scales much better with the number of parameters or participants. This allows the computations to be front-loaded, and can be absorbed by a modeler who then passes off a trained network to other researchers or clinicians seeking to use the model – making neural networks an attractive option for less statistically minded researchers and applied work.

We might also expect the difference between methods to grow as we apply them to models that lack tractable likelihoods. One of the primary appeals of this type of work was that it allowed researchers to more easily explore simulation-based models (Cranmer et al., 2020; Fengler et al., 2021; Kvam et al., 2025). Since we aim to benchmark neural networks against likelihood-based approaches, this is naturally outside the scope of the current work – but the difference in efficiency in the present case will most certainly be exacerbated in the case of simulation-based inference.

### Model comparison

In addition to parameter estimation, trained neural networks can be used for comparing how well different models account for a set of data. The objective of model comparison is to assign a posterior probability to each model that describes how likely it is relative to other models given the data. In Bayesian approaches, the relative credibility of two different models is given by the Bayes factor *BF*, quantifying the relative credibility of one model $M_1$ against another $M_2$ given the data *D*. In most formal model comparisons, we assume no difference in prior probabilities, $Pr(M_1) = Pr(M_2)$, meaning that we don’t believe one model to be more credible than another before evaluating them in light of the data. In this case, the Bayes factor is simply the ratio of the model likelihoods:

6 $$\begin{aligned} BF = \frac{Pr(D| M_1)}{Pr(D|M_2)}. \end{aligned}$$

For *N* models, the relative probability of each model will be proportional to the likelihood of the data multiplied by its prior likelihood. This makes the classification probability equivalent to the posterior probability of a model given by the Bayes factor (Radev et al., 2021).

This approach was applied to the hyperbolic, hyperboloid, and direct difference models. To do so, we generated 100,000 simulated participants from each model, using the same prior values specified above. This yielded 300,000 simulated participants with MCQ data, sampled equally from hyperbolic, hyperboloid, and direct difference models. These 300,000 simulated participants were split into 240,000 for training, 30,000 for validation, and 30,000 for testing, such that each model had 80,000 of its simulations in the training set, 10,000 in the validation set, and 10,000 in the test set.

Next, a neural network was trained to map a set of input data (27 MCQ responses) onto the model that generated the data. This network was identical to the networks used for parameter estimation in the input and hidden layers, with 27 inputs, hidden layers of 100/66/33 nodes, and tanh activation functions. Instead of the parameter values in the output layer, however, the network had a softmax layer that transformed a degree of activation in three output nodes (corresponding to the support for hyperbolic, hyperboloid, and direct difference generative models) into posterior probabilities.

Once the network was trained on the training set, we used the held-out data from the test set to evaluate its ability to discriminate between different generative models. To evaluate the classification network’s predictions, we took the model with the highest posterior probability for each input from the test set and compared it against the true model that was used to generate that simulated participant. The results are shown in Table 2. The network made the correct classification decision approximately 70% of the time. This is well above chance accuracy (33%) and as we show below, better than standard model fit statistics that are commonly used to compare models.

Without a frame of reference, it is difficult to determine whether 70% is good performance or relatively poor. To obtain a benchmark for comparison, we compared the network performance against two model fit statistics that are often used to compare models: the Deviance Information Criterion or DIC (Spiegelhalter et al., 2014), and the Watanabe-Akaike/Widely Available Information Criterion or WAIC (Vehtari et al., 2017).

To evaluate the DIC for each participant, we took the best-fitting group-level priors from the analysis above (Fig. 2), and used them as the priors in a second analysis that estimated the parameters of each model non-hierarchically for each simulated participant. For each simulated participant, we then took the DIC values produced from the model fits in JAGS, and evaluated which model had the lowest DIC for that simulated participant.

**Table 3:** Confusion matrix for DIC-based approach to model comparison

		Inferred model		
		Direct difference	Hyperbolic	Hyperboloid
True model	Direct difference	51.46%	21.36%	27.18%
	Hyperbolic	16.23%	11.01%	72.75%
	Hyperboloid	14.45%	8.09%	77.46%
	Total	95.64%	144.98%	59.38%

**Fig. 3:**
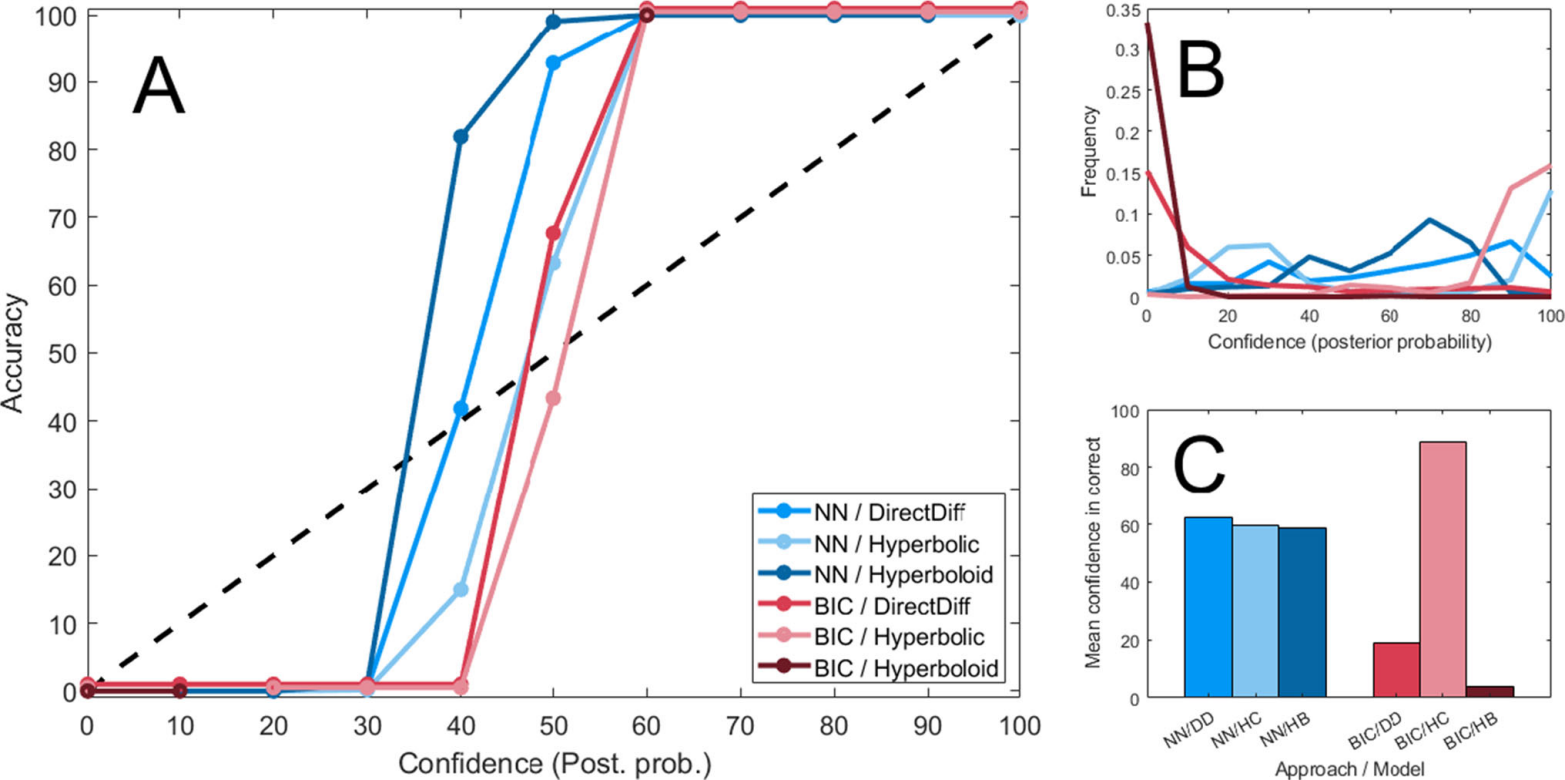
Calibration curves (**A**), relative frequency of certainty levels (**B**), and average posterior probabilities for each model (**C**) based on the output of the classifier neural network [NN] (*blue*) and the Bayesian information criterion [BIC] (*red*). Panel A shows the relationship between posterior probabilities or confidence and the true accuracy at each level of confidence, providing a visual representation of calibration. Panel B shows the relative frequency of different levels of confidence for each approach and model – suggesting that the neural network gave relatively modest levels of confidence while the BIC gave high certainty (0% or 100%) nearly all the time. Panel C shows the average posterior probability assigned to each model when it was the true model – with the neural network being relatively consistent across models and the BIC strongly favoring the hyperbolic model

The model confusion matrix is shown in Table 3. It had a relatively low overall accuracy of only 46.64%, although this tended to occur because it categorized data generated by the hyperbolic model as hyperboloid data. This is a reasonable mistake to make, as the hyperbolic model is nested within the hyperboloid model (when $s = 1$). Its accuracy for the direct difference (51.46%) and for the hyperboloid model (77.46%) were much higher, but its overall performance was inferior to the classification network.

We repeated this process for many alternative model fit metrics, including the log likelihood, BIC, AIC, and WAIC. None of these metrics ultimately outperformed the classification network, and most had similar or worse performance than the DIC. The maximum log likelihood yielded 41% accuracy when inferring the correct model, the BIC (Schwarz, 1978) yielded 37% accuracy, and the WAIC (Vehtari et al., 2017) yielded 51% accuracy. In general, there were no large differences in performance between the models in log likelihoods; as a result, the BIC tended to strongly favor the hyperbolic model while WAIC tended to disfavor the direct difference model in favor of the other two. Overall, the model fit metrics provide some insight into generative processes by identifying the true model with better-than-chance rates, yet they fall far short of the classification network by at least 20% accuracy in almost every case.

#### Calibration

To further evaluate the performance of the classifier network for making inference about what model generated the data, we looked at the posterior probabilities it generated. By default, the outcome of the network is a classification probability, corresponding to its certainty that a particular model was used to generate the data. We can use these probabilities to understand the network’s *calibration*, or how well its confidence or probability corresponds to its accuracy in determining the correct model (Guo et al., 2017).

Likewise, the Bayesian information criterion metric [BIC] can be transformed into a Bayes factor quantifying the relative degree of support for each model under consideration (Kass & Raftery, 1995). The Bayes factor can be used to assign a posterior probability to each model by multiplying by the prior probabilities (each 1/3), allowing us to assess how well calibrated the BIC is as well. This gives us a point of comparison for evaluating the neural network both in terms of calibration and in terms of the distribution of posterior probabilities it produces.

To evaluate the calibration of each approach, we took the posterior probability of each model for each one of 30,000 simulated participants from both the classifier network and the BIC. This posterior probability was then binned into a level of confidence by rounding it to the nearest 10%, resulting in 11 levels of confidence it could express (0%, 10%, 20%, ..., 100%). For each level of confidence, we calculated the actual accuracy (rate at which the true model was correctly identified) for all participants and all models. This allowed us to assess the relationship between the confidence we get from each approach and the degree of confidence we *should* have in the form of the average inference accuracy.

The results of this comparison are shown in Fig. 3, with the neural network shown in blue and the BIC shown in red. Panel A shows the relationship between the posterior probability assigned to a given model (x) and the accuracy or rate with which that model was correctly identified (y). Ideally, a model should fall roughly along the black diagonal line, so that its confidence corresponds closely to its accuracy. Both approaches tended to be underconfident – such that they were more likely to be correct than their confidence would indicate. This effect was slightly stronger for the neural network, where average confidence was lower than average accuracy overall. This is reflected in the average confidence across models (panel C, roughly 60%) being lower than the true accuracy (73%). By contrast, the BIC is relatively well calibrated, with its average confidence (37%) matching its accuracy (37%). However, neither approach perfectly tracked the true accuracy with its (binned) probability or confidence estimates.

It is worth noting that this is a known issue in neural network-based classifiers, such that the probabilities assigned by models do not correlate well to the (binned) accuracy (Mortier et al., 2023). There are now a few methods that might improve the calibration of these probabilities so that they might be more useful as measures of confidence, which is an active research area within machine learning (Guo et al., 2017).

The BIC is extremely inconsistent across the three models, and as we illustrated above, it simply tended to be less accurate. It tends to identify the hyperbolic model as the best-fitting model much more often than it should – likely due to the penalty for the number of parameters overwhelming the evidence in the data/log likelihood. As a result, it yields bimodal posterior probabilities (Fig. 3, panel B) and rarely selects the direct difference or hyperboloid models.

## Application to real data

The parameter recovery and model recovery components have demonstrated that the deep learning approach should, in principle, be able to accurately estimate the parameters of intertemporal choice models as well as recover the best generative model out of a set of candidate models. Next, we apply these approaches to real data to examine how well their conclusions converge and compare with one another. Overall, we demonstrate that the intertemporal choice models can be challenging to fit; however, the main conclusions typically drawn from the parameter estimates using the two approaches are generally similar.

One of the most important domains of application of intertemporal choice models is in addiction, where delay discounting behavior predicts outcomes related to smoking, amphetamine, opioid, and alcohol abuse, among other substances (Bickel et al., 2008; Bickel & Marsch, 2001; Bickel et al., 2012; Kirby et al., 1999a; Yi et al., 2006; Mitchell, 2019; Reynolds, 2006). Novel methods for analyzing this behavioral data, and easily generating model-based insights, have the potential to improve the assessment of substance use problems and accelerate ongoing research on addiction. We therefore apply the approaches developed above to (1) estimate delay discounting and attribute-wise models of intertemporal choice, (2) identify which models best account for behavior, and (3) relate model parameter estimates and best-fitting models to substance-use problems.

**Fig. 4:**
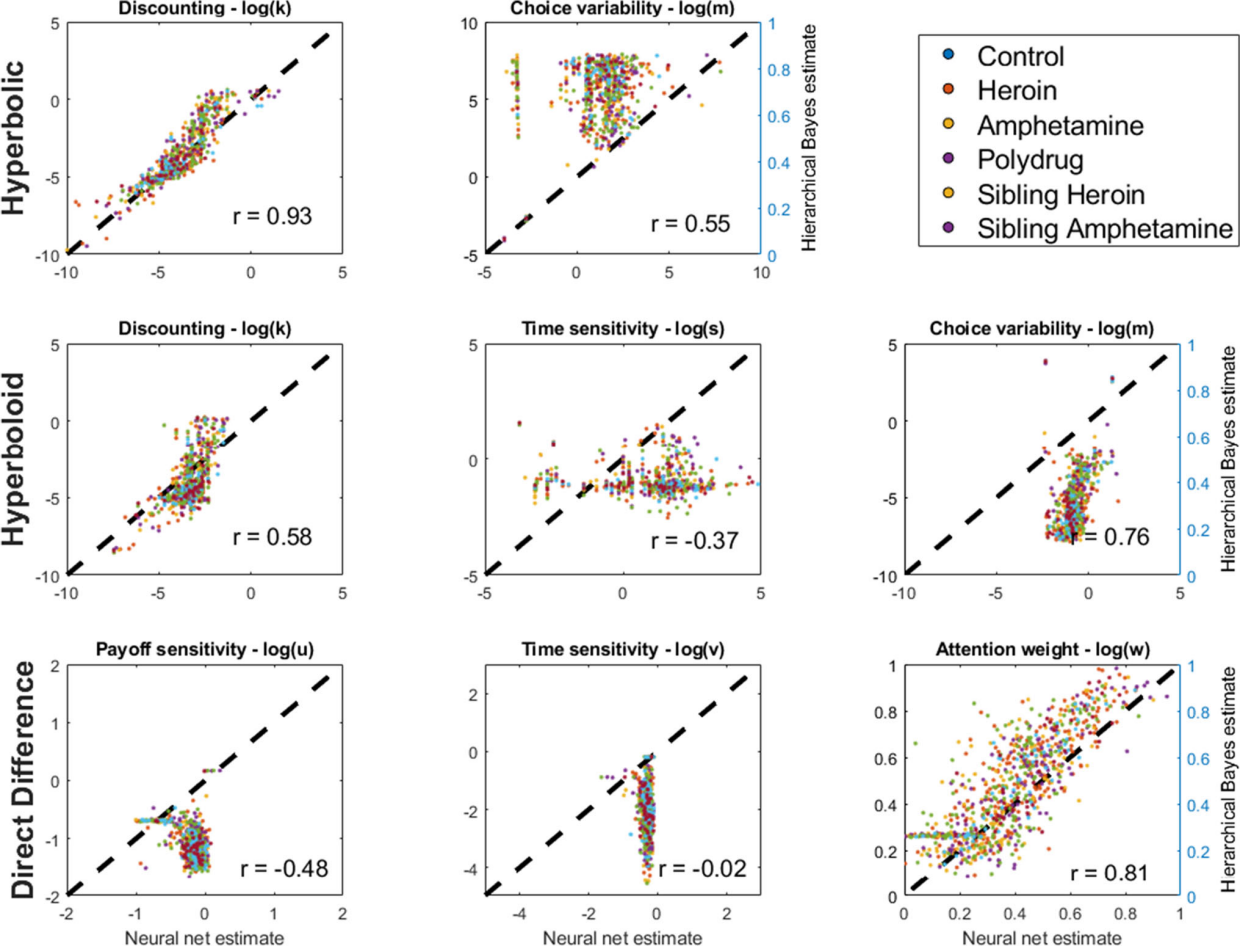
Comparison of parameter estimates generated by a simulation inversion network (*x*) and a hierarchical Bayesian approach (*y*) for each of the parameters of the three models. Participants from different groups are displayed in different *dot colors*. A linear correlation coefficient (*r*) is provided for each plot to assist with understanding the relationship between the two estimation approaches

To do so, we analyze the data from a large data set of intertemporal choice data collected from people with substance use disorders in protracted abstinence (Ahn & Vassileva, 2016; Ahn et al., 2014) as well as matched controls and relatives. This data set also offers the advantage of containing individuals with “pure” substance use problems, i.e., people who have only used amphetamines or heroin, but do not meet the criteria for dependence on other substances. In addition to the substance use populations, these data also include data from the siblings of participants with substance use disorders, providing a secondary set of controls that allow us to evaluate the degree to which behavior affects – versus how it is affected by – substance use outcomes. We included siblings of participants who had heroin use problems and siblings of participants who had amphetamine use problems, for a total of six groups. This allows us to evaluate not only differences between people with and without substance use disorders, but also differences in behavior between different types of substance use and different (pre)dispositions toward substance use.

In total, this data set included 784 participants: 243 healthy controls, 156 amphetamine dependent, 165 heroin dependent, 182 polysubstance-dependent individuals, 67 siblings of individuals with heroin dependence, and 51 siblings of individuals with amphetamine dependence. For each individual in the data set, we used the approaches described above to estimate the parameters of three cognitive models to their data and to classify them according to which model best describes their performance on the monetary choices questionnaire.

### Parameter estimation

The first step of evaluating intertemporal choice behavior in each of the groups of interest was to fit their data using the models described above. For each individual, we passed their behavioral data – binary responses to 27 SS/LL intertemporal choice questions – into a network that was trained to map MCQ data onto model parameters. These were the same networks illustrated above, which were trained based on simulated MCQ data to a high degree of parameter recovery performance.

In addition to the network-based parameter estimates, we also fit the data from each group of participants using a hierarchical Bayesian approach (Molloy et al., 2020). The technical details were the same as the hierarchical Bayesian fitting of the simulated data described above – in terms of the number of samples, burn-ins, chains, and so on – except that all of the data was fit at once rather than in batches of 200. This fitting approach was carried out for each of the substance use groups and models, including hyperbolic, hyperboloid, and direct difference models. Doing so allowed us to evaluate the similarity between neural network and hierarchical Bayesian approaches to parameter estimation. If the two approaches agree, we should see a high degree of mimicry between the two models and convergence between them on parameter estimates.

A plot of the parameter estimates for each individual (dots) for the neural network approach (x) and the maximum a posteriori estimates from the hierarchical Bayesian MCMC approach (y) is shown in Fig. 4. The correlation between estimates for different approaches is presented in the bottom-left of each panel of Fig. 4, providing a measure of the strength of the relationship between estimates for the neural network and hierarchical Bayesian approaches.

The degree of agreement between the two estimation approaches depended heavily on what model was used and what the parameter of interest was. For the discount rate in the hyperbolic model, there was strong agreement between neural networks and Bayesian approaches, as indicated by a nearly perfect correlation between their estimates of the (log) discounting rate – shown at the upper-left of Fig. 4. The correspondence in estimates of choice variability was somewhat lower, at only $r =.55$, but still showed some convergence between estimation methods.

The hyperboloid model (second row of Fig. 4) showed a similarly high degree of convergence between neural network and Bayesian approaches on the discounting and choice variability parameters, indicating that a modeler is likely to make the same conclusions about these parameters regardless of which estimation approach they use for fitting hyperbolic or hyperboloid models. However, estimate correspondence was considerably lower for the time sensitivity parameter; the neural network produced a range of estimates, while the hierarchical Bayesian approach grouped nearly all of the estimates around -1. We suspect this occurred for two reasons. First, there may simply not be enough information in MCQ data to accurately estimate time perception/sensitivity parameters. All of the questions in the MCQ feature a choice between an immediate and a delayed payoff, as opposed to two delayed payoffs. As a result, the form of the time sensitivity function may be hard to estimate. Second, enabled by the lack of information in the data pertaining to time sensitivity, the hierarchical structure of the Bayesian approach resulted in most of the estimates being pulled toward a central tendency of around -1. A lack of information from the data resulted in the group-level distribution carrying greater influence over the individual-level estimates. Consequently, there is relatively little variability across people in the time sensitivity parameter estimates to predict.

There was weaker correspondence between estimation approaches for the direct difference model. While the neural network and hierarchical Bayesian estimates appeared to agree on the attention weight *w* – indexing the degree to which participants paid attention to differences in delays versus differences in payoffs, and shown at the bottom-right of Fig. 4 – they did not agree on the other parameters. This is in large part due to the fact that the neural network predicted very little variability in estimates of time or payoff sensitivity, corresponding to tightly grouped (horizontally) estimates of both parameters. While low variability in the Bayesian approach can be attributed to the multilevel structure, it is not as clearly evident why this occurs with the neural network. We suspect that the neural network simply makes its best guess for time sensitivity, which winds up being close to the mean of its training set – which was approximately $log(v) = -0.15$ or $v =.7$. This behavior would be expected if there was little or no information in the data pertaining to time sensitivity. As we indicated above, all of the pairs of items in MCQ feature one option with no delay, making it difficult to assess time perception. This would explain the performance of the neural network, although not why the hierarchical Bayesian approach provides such wide-spanning estimates.

The point of disagreement between the models comes from payoff sensitivity. There is actually a negative relationship between neural network and hierarchical Bayesian estimates here, which is primarily driven by a few outlying neural network estimates (on the low side) that are estimated to be quite high by the Bayesian method. Below, we investigate the lack of identifiability in these parameters by looking at posterior distributions (from the neural network) for the full versus a reduced direct difference model.

Overall, the neural network and hierarchical Bayesian estimation approaches agree on the estimates of discounting rates and choice variability – whether for the hyperbolic or hyperboloid models – and the attention weight parameter in the direct difference model. Interestingly, these are the model parameters that are typically considered the most “important” – as they correspond closely to a decision-maker’s preference for smaller sooner outcomes or larger later ones. From the standpoint of clinical predictions, which are based mainly on discounting rates, either estimation approach should produce reasonable results.

Care should be taken when using the direct difference model in general, as we suggested in the parameter recovery section above. This model may not be well suited to modeling data from the MCQ, either because its structure needs more data or different types of comparisons to be accurately fit. Given the model’s emphasis on differences, it may simply be that the stimuli from the MCQ are simply not conducive to good estimates from the direct difference model. Both neural network and hierarchical Bayesian approaches attempt to extract as much information as possible from the data, so there is good reason to think that the 27 bits of information provided by the MCQ limit the potential accuracy of both approaches. This hypothesis is supported by the limited recoverability of the models – and especially the direct difference model shown in Fig. 2. Other tasks may help us better understand the limitations of the stimuli versus the limitations of the estimation approach. In the next section, we address this question by fitting many different configurations of stimuli with both approaches and by looking at the individual-level posterior distributions provided by a posterior sampling approach. In general, time sensitivity is difficult to recover from intertemporal choice data, indicating that while time perception might be a useful construct for understanding choice (Grondin, 2010; Killeen & Grondin, 2021), it may be difficult to assess with choice data alone. Instead, secondary tasks may be necessary to assess time perception before its impact on choice is apparent (Chen & Zhao, 2024).

**Fig. 5:**
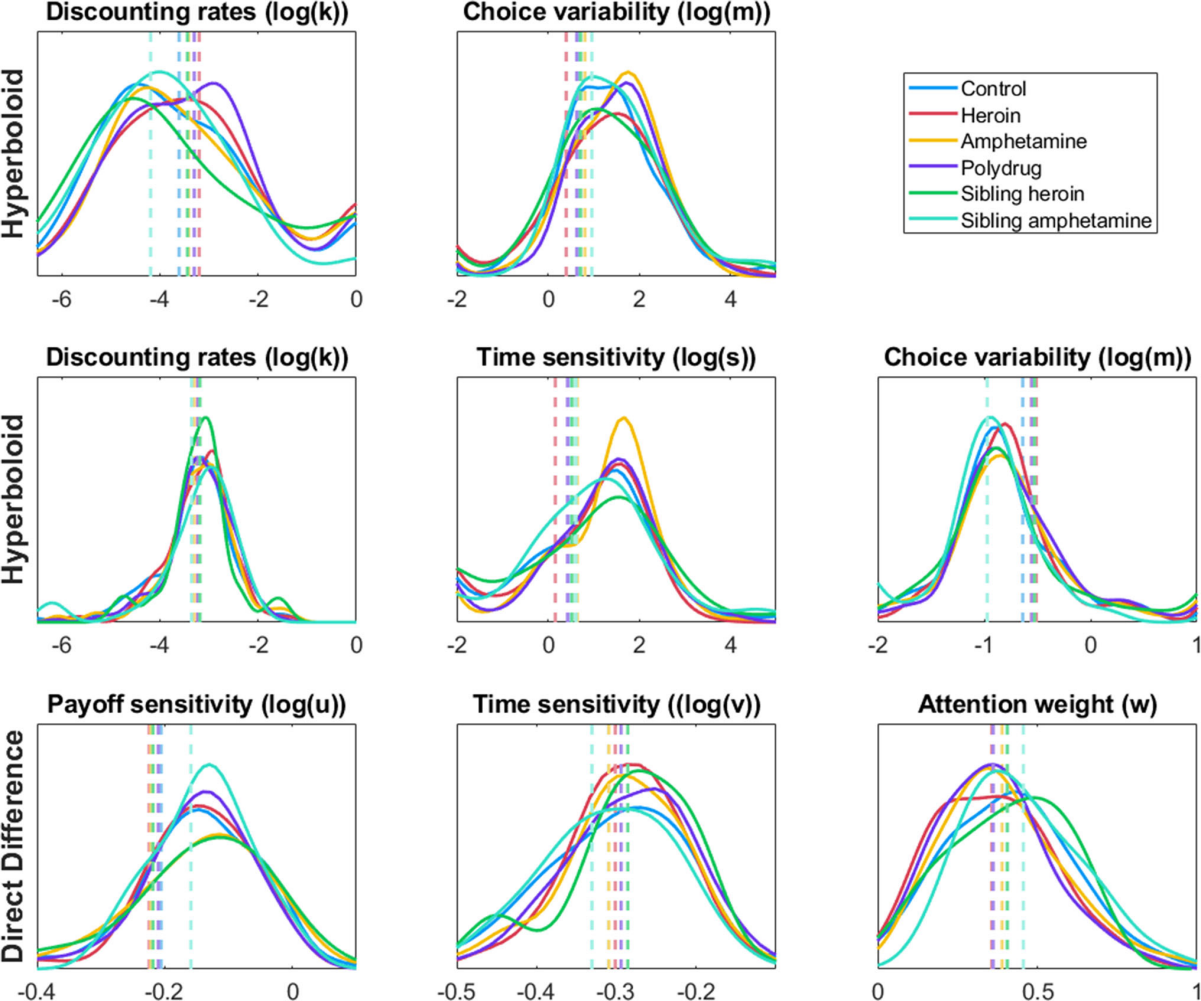
Parameter estimates for each parameter of the hyperbolic discounting (*top*), hyperboloid (*middle*), and direct difference (*bottom*) models, fit using the simulation inversion networks. Each substance use group is shown in a different color. Distributions illustrate estimated probability density for each group, normalized by the size of the group, and *dotted vertical lines* correspond to the mean estimate for that group and parameter

#### Group-level comparisons

Once we have obtained parameter estimates for each participant in the study, we can compare the results for each of the different groups. The distributions of estimates for each parameter and each substance use group are shown in Fig. 4, with the control group shown in blue, pure heroin use group in red, pure amphetamine use group in yellow, polysubstance use group in purple, heroin use siblings in green, and amphetamine use siblings in cyan. Each plot shows the relative frequency of each group at each level of the parameters, based on the neural network estimates. The control group, being the largest, is naturally the highest overall in each plot. However, at certain levels of model parameters – such as high levels of discounting ($\log (k)$) or low levels of attention to payoff ($\log (w)$) – it becomes more likely that a participant with that level of impulsivity or attention weight actually belongs to one of the other groups (Fig. 5).

The central tendency of each group is shown as a dotted vertical line in the corresponding color. The three different models appear to identify several differences between the groups. Specifically, both the hyperbolic and hyperboloid models seem to indicate that the control group, and to a lesser extent the siblings of heroin and amphetamine users, show lower rates of temporal discounting – a Bayesian ANOVA indicates weak evidence for differences among the groups for both hyperbolic discounting rate (BF = 1.45) and hyperboloid discounting rate (BF = 2.06). While certainly not conclusive, this finding corresponds well to previous literature indicating that people with substance use problems discount future outcomes at a steeper rate than those without substance use problems (Kirby et al., 1999a; Bickel & Marsch, 2001; Bickel et al., 2012; Vassileva & Conrod, 2018; Ahn et al., 2014).

The direct difference model seems to attribute differences between the groups to slightly different processes. In particular, these parameter estimates indicate greater weight or attention to payoffs – in the form of higher estimates of the *w* parameters – in the control group relative to the others. As before, a Bayesian ANOVA indicates weak support for differences between the groups on this parameter (BF = 2.92). This aligns with previous work on the direct difference model using a similar data set (Kvam et al., 2021), where the attention weight parameter drove differences in delay discounting behavior between substance use and control groups.

It is worth a short note that differences between groups were found for the parameters that could most reliably be recovered (see Fig. 2). It is possible that there are differences in time perception, choice variability, or payoff sensitivity that are simply too difficult to pick up with the MCQ data and the sample sizes we use here. Richer data, and perhaps better models that leverage process-level information on decisions, can of course shed light on additional differences in behavior between groups.

### Model comparison

In addition to detecting differences in behavior via parameter estimates, the neural network approach can also assign posterior likelihoods to different models in order to identify whose data is best fit by which model. One reason that we observe differences in discounting behavior between groups could be that participants actually implement different strategies for intertemporal decision-making. For example, one group might favor an alternative-based approach to choice while others favor an approach where they contrast the attributes of their options in order to come up with a balance of support between the options (Cheng & González-Vallejo, 2016; Lee & Hare, 2023). These approaches would likely lead to the hyperbolic/hyperboloid or direct difference models fitting better, respectively.

The interaction between choice strategy and substance use outcomes has not been thoroughly explored before. It is possible that apparent differences in discounting or attention are actually driven by differences in choice strategies for comparing and contrasting the available options. This can be inferred from visual fixations (Amasino et al., 2019; Franco-Watkins et al., 2016) or from model comparisons (Cheng & González-Vallejo, 2016). Here, we carry out the latter using an additional neural network trained to identify differences in generative model from observed behavior (see Table 2).

Overall, the model comparison yielded a total of 215 participants who were best fit by the direct difference model, 550 participants who were best fit by the hyperbolic discounting model, and only 99 who were best fit by the hyperboloid model. In some ways, this runs counter to previous results that tended to favor the direct difference model or hyperboloid over the hyperbolic model (Dai & Busemeyer, 2014; Cheng & González-Vallejo, 2016; Lee & Hare, 2023; Myerson & Green, 1995; Rachlin, 2006). However, it is less surprising in light of the observation that these fits are based on only 27 binary choices, rather than a larger set of stimuli where payoffs and delays were more widely and systematically varied. Because there is relatively little data to constrain the models, the model comparison naturally favors a simpler, more parsimonious model (hyperbolic). We expect that richer data incorporating response times (Dai et al., 2018), eyetracking (Amasino et al., 2019; Franco-Watkins et al., 2016; Lee & Hare, 2023) or more trials would potentially reverse these conclusions.

As for the relationship between choice strategy and substance use groups, we found nothing of interest. The probabilities of being assigned to one more or another had no relationship to which group a participant came from (all Bayes factors > 100 in favor of the null). While there may be interesting differences among groups in terms of the model parameter estimates, the best-fitting model did not appear to be predictive of the substance use group.

## Input structures and posterior sampling

The results we have presented so far have focused on benchmarking existing approaches to likelihood-free parameter estimation and model comparison against existing likelihood-based approaches like Bayesian MCMC. These have examined cases that play to the strengths of neural networks, in that the MCQ has a fixed set of inputs and we sought point estimates of individual differences. In this section, we next focus on cases where neural networks have not been used as much. Specifically, we aim to (a) apply them to cases where the inputs (stimuli, trials) are not fixed, and (b) quantify the uncertainty in their estimates. This will address two of the biggest criticisms of neural networks thus far; namely, that they are unsuitable for complex experimental designs and that they are unable to provide a complete joint posterior estimate of parameters.

As it turns out, neural networks can actually accomplish both of these. In the first part of this section, we examine how recurrent layers in a neural network allow it to deal with inputs of varying lengths, making it adaptable to different stimuli and trial numbers (Rmus et al., 2023; Lueckmann et al., 2017). In the second part of the section, we examine how dropout layers can be used to vary the neural network’s predictions and obtain a complete joint posterior distribution over parameter values (Gal & Ghahramani, 2016).

We note that both posterior sampling and recurrent networks have been presented in previous work (Radev et al., 2020, 2021; Gonçalves et al., 2020; Lueckmann et al., 2017). However, they remain central criticisms levied against deep learning approaches to parameter estimation, in part because these methods are still new to psychology. To elucidate how neural networks can be used to handle different experimental designs, we next examine these approaches and apply them to modeling intertemporal choice.

### Flexible inputs for flexible designs

The first major limitation to the neural network approach we have adopted thus far is that it requires a fixed set of inputs to the neural network. For the monetary choice questionnaire, this is no concern, as every participant completes the same set of 27 items, consistently yielding 27 inputs to the network. However, a fixed set of inputs prevents the network from being able to fit different stimuli, numbers of trials, or other variations in experimental paradigms. As we have shown, the particular set of stimuli in the MCQ are not comprehensive, and show some weaknesses when it comes to fitting certain parameters and certain models. For example, we struggled to estimate the time sensitivity parameters in both the hyperboloid and direct difference models. This may simply be due to the lack of variation in the delay of the smaller-sooner outcome, meaning that all of the time sensitivity must be estimated based on the manipulations of the larger-later delay.

Many approaches to intertemporal choice use different paradigms with different sets of stimuli. There are a variety of intertemporal choice experiments that one could use to assess impulsivity, ranging from staircasing procedures that systematically adjust the outcome or delay until indifference points are identified (Mazur, 1987; Sawicki & Białek, 2016; Rodriguez et al., 2014; Myerson & Green, 1995) to choice procedures where random or fixed sets of stimuli are presented to assess decisions across a variety of combinations of payoffs and delays (Kvam et al., 2023; Loewenstein & Thaler, 1989; Dai & Busemeyer, 2014). Even more challenging are adaptive experimental designs where the choices a participant makes determine the subsequent stimuli that they are presented, rather than simply stopping when they change their minds (Cavagnaro et al., 2016). Creating a tool that can handle these different types of experiments necessitates making the input to the network more flexible.

There are a variety of ways to address this problem. The first possibility is to allow for a sequence input to a neural network, with a five-channel “signal” describing the payoff for the SS option, the payoff for the LL option, the delay for the SS option, the delay for the LL option, and a participant’s response to that stimulus (e.g., 1 = chose LL, -1 = chose SS). This type of input can be fed into a neural network using a recurrent layer (Rmus et al., 2023) that turns a temporal sequence of data into a fixed number of output nodes that can then be fed into the rest of the network. In other cases, the input can be “padded” with zeros so that it is always a fixed length, no matter how many trials a participant completed.

Alternatively, the data can be summarized in some way using sufficient statistics to describe behavior across the entirety of the task. Rather than feeding in a fixed list of responses, the underlying data are summarized using a consistent set of metrics that describe how choices and uncertainty change across combinations of smaller-sooner stimuli and larger-later stimuli. We test the recurrent network method next, again comparing it to hierarchical Bayesian methods that can readily adapt to different experimental designs. In the supplement, we also provide a method that uses a generalized input structure to sidestep the issue of varying stimuli. However, we generally recommend the recurrent network approach as it is simpler, less computationally intensive, and equally (or often, more) effective than the generalized-input grid method presented in the supplement.

#### Recurrent networks

The first approach we examine is based on recurrent neural networks that allow a sequence of inputs to the neural network to be mapped onto a fixed set of output nodes (sequence-to-last). Specifically, we use a type of recurrent layer called a long-short-term memory or LSTM layer. This allows a recurrent neural network to keep track of previous information (about early trials/stimuli) while sequentially reading in newer information (about later stimuli/behavior) before passing information about the inputs onto the next layer of the network. This approach treats performance on an intertemporal choice task as a 5-channel sequence of observations, where four channels describe the stimuli and one channel describes the decision a participant makes in response to those stimuli. This approach theoretically allows for time-varying parameters (Schumacher et al., 2023); however, the critical function it plays here is that it allows for sequences of varying lengths to serve as inputs to the neural network, permitting the number of trials and the stimuli to vary freely.

To test this approach, we generated 100,000 simulated data sets from each model, with each data set varying from 20 to 200 trials in length (drawn at random from a uniform distribution). To create the stimuli in each data set, we drew a variety of stimulus values for the smaller-sooner and larger-later options. The payoff for the smaller-sooner option was drawn first from a uniform distribution as $SS \sim Uniform(5,100)$. Next, the payoff for the larger-later option was drawn from a distribution that depended on the payoff of the smaller-sooner option (so that it could be larger), as $LL \sim SS + Gamma(2,15)$. Next, we drew a delay for the smaller sooner option from a gamma distribution, $t_{SS} \sim Gamma(1,30)$. Likewise, the delay for the larger-later option was fixed to be larger than that of the smaller-sooner option, set as $t_{LL} \sim t_{SS} + Gamma(2,30)$. This yielded stimuli that roughly mimicked the values and differences among stimuli in the Monetary Choice Questionnaire, providing sets of nontrivial but sufficiently variable choice problems.

This approach to simulating data is based on participants who see independent, identically distributed combinations of stimuli across trials of the experiment. However, the input structure should work equally well for stimuli that are sampled using a staircasing procedure (Mazur, 1987) or stimuli that are generated from adaptive experimental procedures like adaptive design optimization (Cavagnaro et al., 2010, 2016). So while we used randomly generated, non-adaptive stimuli for training, there is no reason to believe this approach would not work for other experimental structures.

#### Results

The network we used had a single sequence input layer followed by an LSTM layer with 150 recurrent nodes. The final state of the LSTM layer was then mapped onto another hidden layer with 100 nodes and a ReLU activation function. This was then passed through two more fully connected hidden layers with 80 and 30 nodes, and finally into a regression layer. We used a squared-error loss function and ADAM algorithm for updating the neural network weights (Kingma & Ba, 2014) across 50 epochs. The input data were split into 80,000 for the training set and 20,000 for the validation set.

**Fig. 6:**
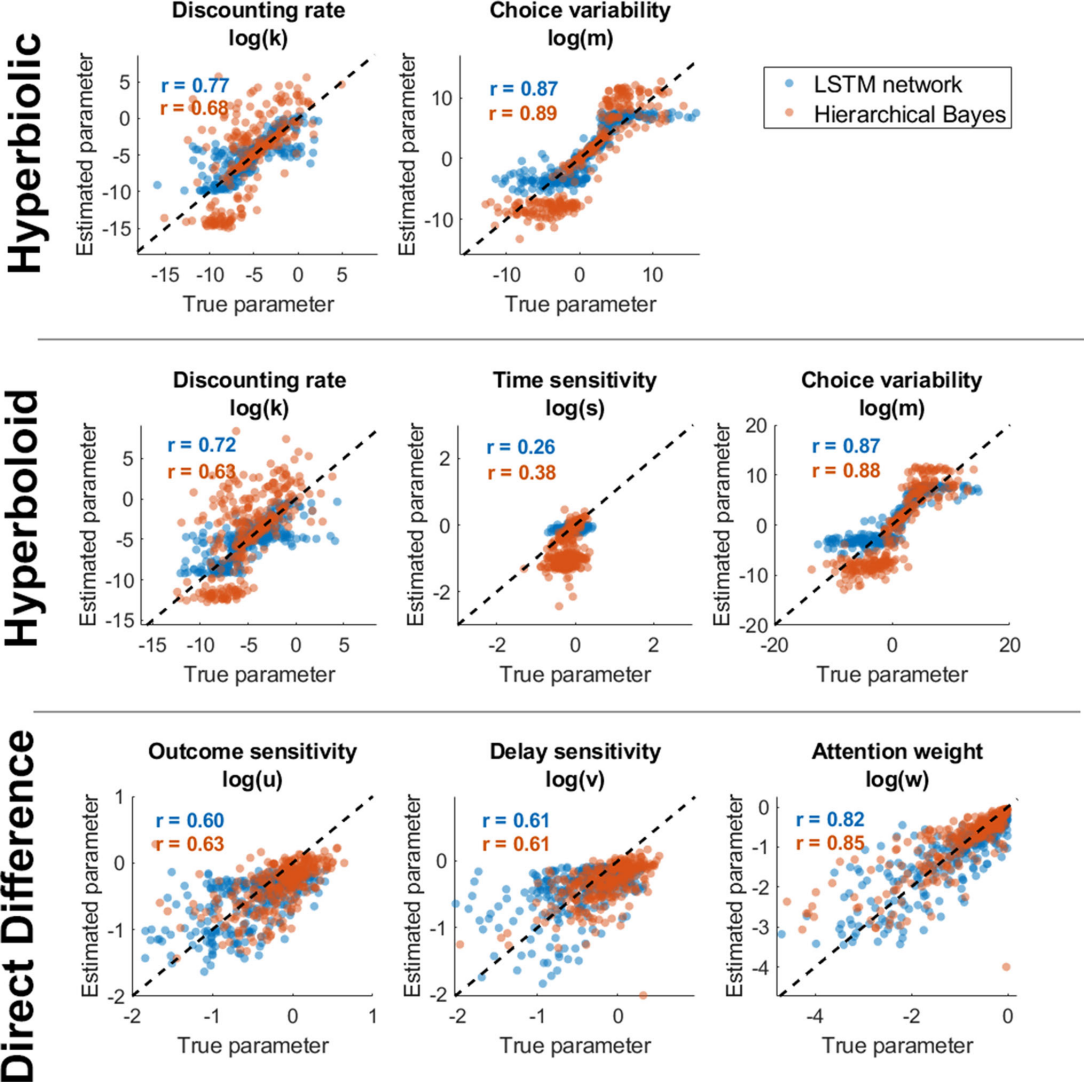
Relationship between true (*x*) and estimated (*y*) parameter values for estimates generated by the recurrent/LSTM neural network for each model and parameter. Each row corresponds to a different model (*top* = hyperbolic, *middle* = hyperboloid, *bottom* = direct difference). *Blue dots* correspond to example predictions from the LSTM network, while *orange dots* correspond to predictions from a hierarchical Bayesian estimate of each parameter

After training, we evaluated the performance of the neural network on recovering the parameters of the model that was used to generate the data in the first place, using its performance on the validation data in order to avoid overfitting (although validation and training loss were nearly identical). The results for each of the models, shown as a plot of the true parameters used to generate each data set (x) against the values estimated by the recurrent network (y), are shown in Fig. 6.

The performance of this network was compared against that of a hierarchical Bayesian model implemented in JAGS, where each parameter had hyperpriors specifying the prior probabilities of different values of the group-level parameters. This approach was carried out in the same way as the hierarchical Bayesian models presented above (Fig. 2), using the same priors and same modeling code. The hierarchical Bayesian method used likelihoods and was already flexible to the number of data points, meaning there were no changes needed between the MCQ and the data sets here that could vary in terms of the stimuli and number of trials. The question at hand was whether the LSTM approach could mimic the performance of the Bayesian approach, which is flexible by default and provides optimal posterior inferences for the available data.

The results are shown in Fig. 6. In general, the recurrent network did a good job of recovering the true parameters that were used to create each data set. Because the stimuli were randomly generated, there will naturally be some sets of stimuli that are either too small or not diagnostic of the model parameters. Because the mapping between parameters and observations is stochastic, perfect performance is impossible.

The LSTM network predictions, shown in blue, are consistently performing to the high standard set by the hierarchical Bayesian estimation method, shown in orange. For each of the models and each of their parameters, there are almost no differences in performance between the two estimation methods. There are some trade-offs for the hyperboloid model – where the LSTM tends to better estimate discounting rate, while the hierarchical Bayesian approach tends to better estimate time sensitivity. However, even these trade-offs are fairly minor and do not strongly tip the balance one way or another.

One observation worth noting is that neither approach does very well at estimating the time sensitivity parameters. Despite having more data, and more stimuli with delays (with many SS options having a delay, rather than being immediate), there was not a noticeable improvement in how well the neural network or hierarchical Bayesian methods could recover time sensitivity ($\log (s)$) in the hyperboloid model. There were typically around 8 times the number of delays being present in the LSTM training data sets compared to the MCQ training sets, both because the SS options were delayed (2$\times$ the delays) and because there was an average of 110 trials (20-200) rather than just 27 (4$\times$ the trials). Theoretically, this should make time sensitivity easier to estimate, but there was no real difference between the MCQ and variable-stimuli data. There may be some combinations of stimuli that allow for finer resolution of time perception, or possibly different tasks that could be leveraged to independently constrain time sensitivity (Chen & Zhao, 2024). However, our present results suggest that this parameter will be difficult to estimate even with a variety of stimuli.

**Table 4:** Confusion matrix for recurrent LSTM neural network-based approach to model comparison. Rows correspond to the generative model from which the data were simulated (True model), and columns correspond to the percentage of data from each generative model classified into each category/model (Inferred model). Rows naturally sum to 100%, while marginal totals at the bottom correspond to the relative bias to categorize the model as direct difference, hyperbolic, or hyperboloid

		Inferred model		
		Direct difference	Hyperbolic	Hyperboloid
True model	Direct difference	96.34%	1.98%	1.68%
	Hyperbolic	2.69%	96.06%	1.24%
	Hyperboloid	2.73%	0.59%	96.68%
	Total	101.76%	98.63%	99.60%

In addition to parameter recovery, we also examined how well the network could carry out model recovery by classifying input sequences according to which model was used to generate them. The structure of this network was identical to the ones used to estimate model parameters with the exception of the final layers, which included a softmax layer and a classification layer (to make its output a category/model identification) in place of a regression layer (previously used for parameter estimation).

As with the MCQ, the model recovery network with an LSTM to handle variable inputs performed extremely well, with an overall accuracy of over 96%. The results of the model recovery study, and specifically its predictions for the validation set on which it was not trained, are shown in Table 4. As shown, there was not a particularly strong bias toward any single model – there were slightly more predictions favoring the direct difference model, but mistakes still happened no more than 3% of the time.

### Quantifying uncertainty

A strength of traditional Bayesian approaches using MCMC is their ability to characterize the uncertainty about parameter values using a joint posterior distribution. Previous approaches to quantifying uncertainty in neural networks have typically been used to find a best point estimate, and then trained to estimate the magnitude of the difference between this estimate and the true value (Radev et al., 2020; Sokratous et al., 2023). In networks trained to estimate the parameters of a cognitive model, this effectively operates as a maximum a posteriori [MAP] estimate and posterior variance on each parameter.

Critically, reducing parameter estimates to a mean and variance ignores the covariance structure of the parameter estimates in the posterior. This has drawn focus to likelihood-approximation networks in place of direct estimation networks, where neural networks are used to compute a single likelihood and then fed into an MCMC process that actually draws posterior samples (Fengler et al., 2021). While effective, this approach still requires additional methods for posterior sampling, making it substantially less efficient than direct simulation inversion networks we use here.

Fortunately, it is possible to modify the structure of simulation inversion networks in a simple way to produce posterior estimates. There are several approaches in simulation-based inference and amortized Bayesian inference that allow for fully Bayesian inference (Cranmer et al., 2020). Here, we focus on work by Gal and Ghahramani (2016), who showed that it is possible to draw Bayesian posterior samples from a trained neural network by adding dropout layers. In a neural network, a dropout layer randomly sets some proportion of the nodes (and thus their weights to the next layer) to zero, effectively knocking them out for that iteration of training or testing (Baldi & Sadowski, 2013; Kvam et al., in press). This serves two functions. First, it allows the neural network to make robust and generalizable predictions: by removing certain paths through the network, it forces the training process to pass information through many different routes that will all converge on similar destinations. This helps reduce overfitting to the training set, bringing training performance closer to validation performance.

**Fig. 7:**
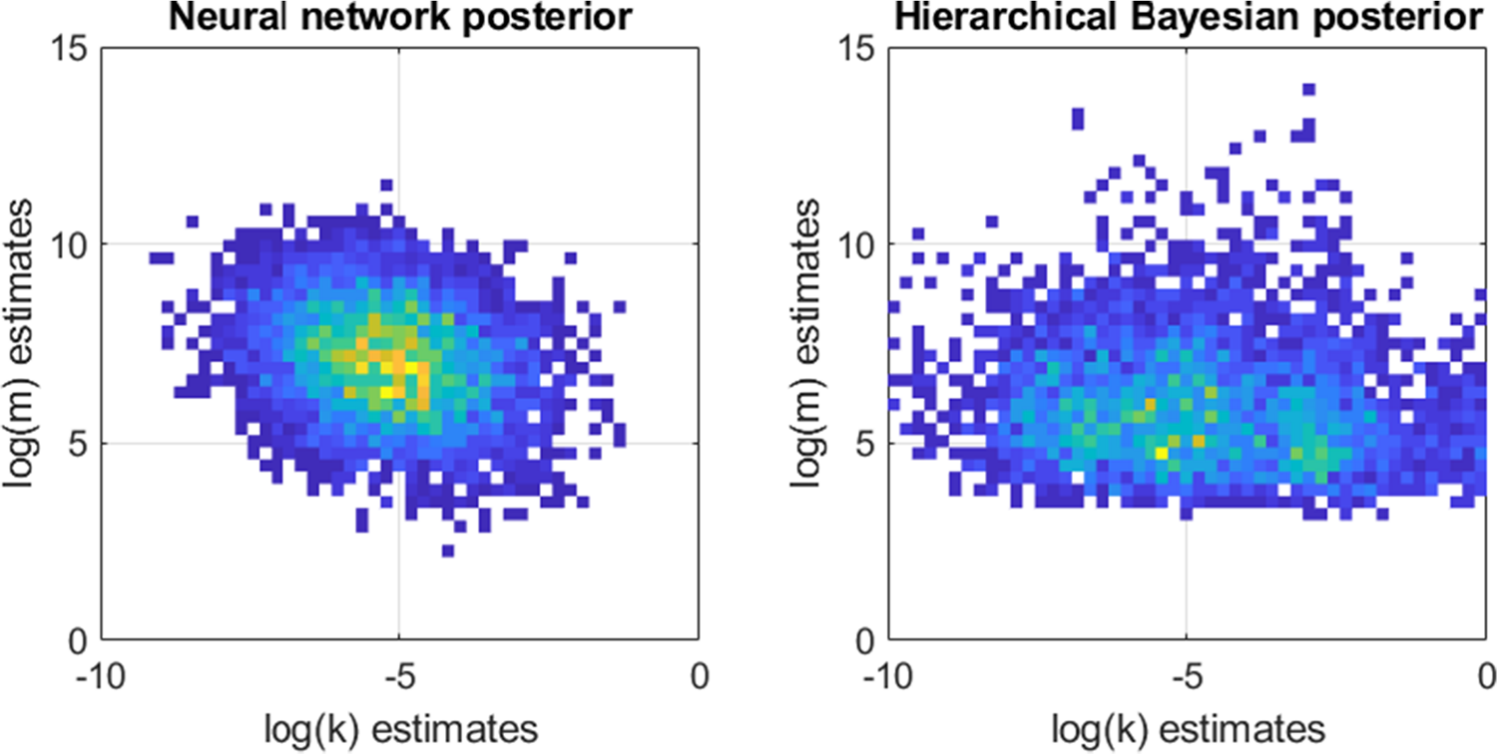
Posterior distribution over $\log (k)$ (*x*) and $\log (m)$ (*y*) parameters for an example participant, shown for the neural network PDGP generated from dropout samples (*left*) and hierarchical Bayesian estimates from MCMC samples (*right*)

Second, and more important to the question of posterior estimation, a dropout layer allows a neural network to vary its predictions from iteration to iteration by passing information through multiple different paths to the outputs. By randomly dropping different nodes in the network, we can apply the network to the same data many times and get different results each time. As Gal and Ghahramani (2016) show, doing so approximates a Bayesian sampling process, where each output of the neural network (with random dropout) constitutes a single draw from a posterior distribution of a Gaussian process. By drawing many predictions from the network for the same input, we create a probability distribution over possible outputs. Formally, we refer to this as the posterior of a deep Gaussian process, or PDGP for short.

This method of sampling from the posterior is much more efficient than traditional MCMC samplers like Metropolis-Hastings, Gibbs, NUTS, slice, Hamiltonian, or even importance sampling (Gelfand, 2000; Neal, 2003; Neal et al., 2011; Hoffman et al., 2014) because it does not require computing the likelihood or prior on each sampling step. Instead, it only requires evaluation through the network, which can be parallelized and implemented on a GPU (Oh & Jung, 2004). As we show in Table 1, this can create quite significant differences in overall model fitting time, especially when the number of parameters to estimate becomes large.

To test this approach, we added a dropout layer to the penultimate layer of the simulation inversion networks from the MCQ above. Specifically, each node in the fourth layer of each network was set to zero with a probability of .5 during each step of training and each application to the data. For the real data, we applied the network 5000 times to generate a PDGP distribution over each parameter for each model and each participant. This was compared against a hierarchical Bayesian model of the data, which also featured 5000 draws from the posterior distribution (using a single chain, since convergence was established already).

Note that unlike the MCMC chain, a PDGP distribution generated by the neural network does not have any sequential dependency, resulting in a greater number of “effective samples” due to autocorrelation in the MCMC chain (Kruschke, 2014; Vehtari et al., 2021). Furthermore, it does not require a burn-in process, because there is no initial location or sample that can systematically diverge from the high-density regions of the posterior. As a result, the MCMC process requires more samples to create a posterior than the neural network due to both autocorrelation and burn-in sampling.

As we indicated above, this is not the only way to quantify uncertainty in a Bayesian way using neural networks. Neural Bayes estimators, Bayesian neural networks, and normalizing flows can produce distributions over potential outputs that approximate similar sorts of posterior distributions over network parameters (Sainsbury-Dale et al., 2024; Lenzi et al., 2023). However, it is a simple and convenient way to efficiently generate a posterior using a method (dropout layers) that is already commonly incorporated into neural networks and straightforward to implement.

**Fig. 8:**
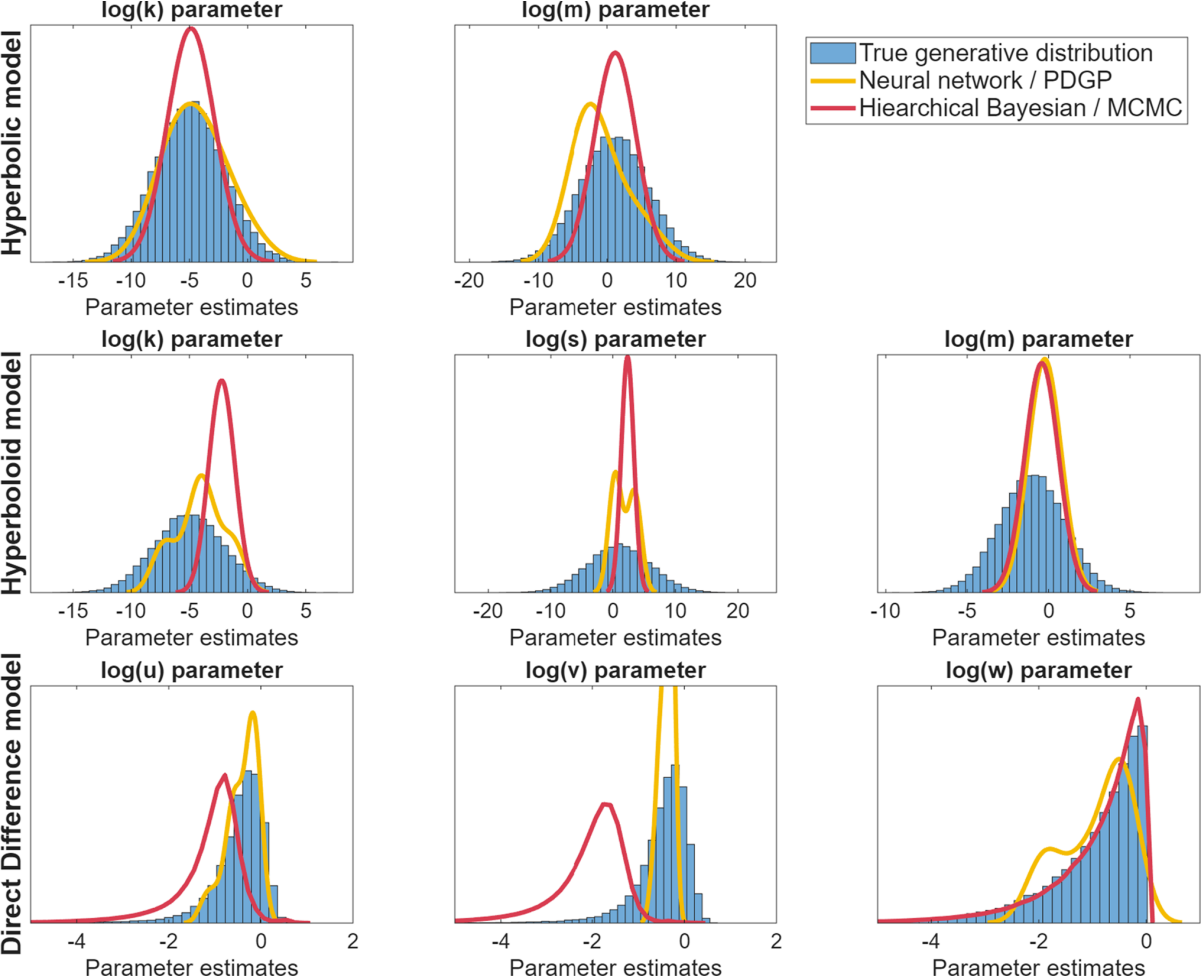
True group-level distributions (*histogram*) compared against the posterior distributions generated from the neural network (*yellow*) and hierarchical Bayesian MCMC samples (*red*), for each different model (*rows*) and parameter (*columns*). Posterior distributions for the neural network and hierarchical Bayesian approaches depict a smoothed kernel density estimate of the corresponding set of posterior samples

#### Results

An example of the posterior distribution of parameter estimates for a single participant, including the PDGP derived from the neural network and a posterior derived from the hierarchical Bayesian procedure, is shown in Fig. 7. There are several important points that this illustrates. First, the neural network is not simply learning the posterior mean and variance for each parameter and combining the marginal distributions. Rather, the (slight negative) covariance between the two parameters shown on the left of Fig. 7 indicates that it is, in fact, constructing a joint posterior distribution. This overcomes a key limitation of previous neural network parameter estimation approaches, in that it produces a complete joint posterior rather than marginal means and variances.

A second thing to note is that the neural network and hierarchical Bayesian approaches largely agree on the distributions of $\log (k)$ and $\log (m)$, again indicating convergence in not only the best estimates but also the posterior. The hierarchical Bayesian approach appears to be bounded somewhat on the lower end, indicating perhaps that the group-level constraints make it unlikely to observe $\log (m)$ estimates lower than 3 for this type of data, whereas the neural network approximates a Gaussian distribution.

*Efficiency.* This can be carried out for any simulated participant in the data set to obtain posterior estimates from either the neural network PDGP or hierarchical Bayesian procedure. However, in our experience, the neural network tends to be much faster. The time to extract 5000 samples from each approach, for 300 and 3000 participants, is provided in Table 1. These timings are based on a single run each and should not be taken as an exhaustive and precise estimate of the timing of either approach. However, a speed-up of over two orders of magnitude is highly suggestive regarding the efficiency of sampling from a neural network versus sampling using an MCMC process.

*Group-level posteriors.* The time it takes to run the neural network or MCMC chain is only one component of the posterior sampling process. More often, we are interested in accurately representing the uncertainty about individual- or group-level estimates. To evaluate how well each approach characterized the group-level estimates, we generated a new synthetic data set of 300 participants using the same priors described above for the MCQ data. Next, we obtained 5000 posterior samples from the trained dropout neural network and 5000 posterior samples from a hierarchical Bayesian MCMC process (using JAGS). For each one, we then computed (or directly used the group-level mean, in the case of the hierarchical model) the mean across subjects of each sample for each parameter. This allowed us to create a posterior distribution of the group-level estimates of each parameter.

This group-level distribution was computed for each of the parameters of the hyperbolic, hyperboloid, and direct difference models. Because we used synthetic participants, we were able to compare the neural network and hierarchical Bayesian approaches against the ground-truth group-level distributions for each parameter. These are shown in Fig. 8.

In general, both models did fairly well at recovering the group-level posterior distributions of the hyperbolic and hyperboloid models, with the exception of the $\log (m)$ parameter, where both approaches drastically over-estimated the posterior variance. For the direct difference model, the neural network slightly underestimated the posterior variance of the $\log (u)$ and $\log (v)$ parameters, while the hierarchical Bayesian approach slightly underestimated them. Both approaches did significantly better with the $\log (w)$ parameter, although the hierarchical Bayesian posterior was substantially closer than the neural network.

Critical to note is that the neural network dropout-sampling approach approximates a Gaussian process, meaning that the posterior distribution for each individual will tend to resemble a multivariate-normal. The group-level distribution will then be a mixture of Gaussians. As a result, skew in the posterior distributions (like in the bottom-right panel of Fig. 8) result from some participants having lower values and others having higher values of the estimated parameter. By contrast, the hierarchical Bayesian approach favors a normal distribution of parameters across participants due to the constraints of the hyperpriors (i.e., it constraints individual-level estimates using a normal likelihood) but it does not assume a particular form of the posterior. Therefore, it can create skewed distributions of posteriors like in the bottom panels, but is still constrained by the likelihoods used for the shape of the group-level distribution (Kruschke, 2014).

Overall, we see the differences between the two approaches in producing accurate posteriors as not too substantial. Each method misses in different ways for different parameters, and for slightly different reasons, but at least still corresponds to the central tendency of the true distributions.

**Fig. 9:**
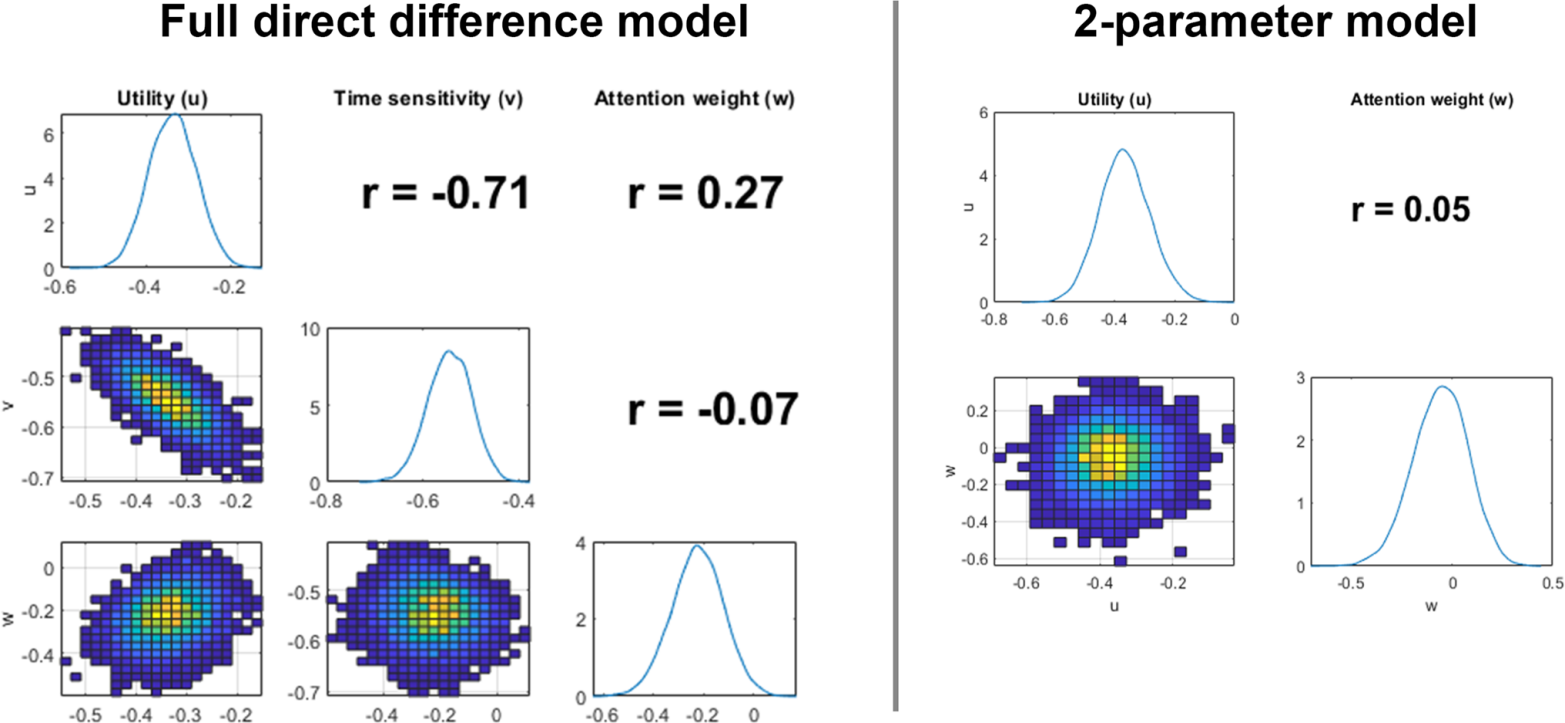
Posterior parameter estimates for the full direct difference model (*left*) and for a restricted version of the model with time sensitivity fixed at 0.7 (*right*). The diagonal plots show the marginal posterior distribution of each parameter for a single participant, while the off-diagonal plots show the correlations between different parameters – with lower diagonal showing a density plot of the posterior samples from the neural network, and the upper diagonals showing their linear correlation

Another way to use the posterior estimates is to evaluate cases where there are strong correlations between samples for different parameter estimates. In cases like the direct difference model (Figs. 2 and 6), it may be difficult to estimate one parameter as a result of including another due to overparameterization. For example, the difficulties in estimating time sensitivity might occur because payoff sensitivity is included in the model, or vice versa. To identify when this happens, a modeler can look at the shape of the posterior distribution, looking for instances where there are strong correlations between samples for different parameters.

To investigate this possibility and demonstrate how the joint posterior derived from the neural network can be used in this way, we present a joint posterior for the direct difference model for an example participant in Fig. 9. As shown on the left side, there are strong relationships between all of the model parameters, making it exceptionally difficult to estimate any single one of them. This indicates a problem with the combination of experimental paradigm and model, as the stimuli are not sufficient to separately identify all three model parameters. In particular, the strong correlations between *u* and *v* make it difficult to estimate either one.

We can also use the joint posterior to rectify this problem, examining the effect of fixing one of the model parameters while estimating the others freely. The effect of doing so is illustrated on the right side of Fig. 9, where the time sensitivity parameter is set to a value close to the group-level mean ($v = 0.7$). When this modification is introduced, we eliminate the strong correlation between *u* and *v* and the remaining correlation between *u* and *w* essentially disappears.

Although it does not appear to affect the marginal posterior distributions here, this may also help with estimating the remaining parameters of an overparameterized model by reducing the uncertainty introduced by interfering parameters. Naturally, choosing which parameter to fix and understanding the effect or limitations on insights that doing so imposes is a difficult problem. This modeling choice remains up to the modeler – but the neural network joint posterior can at least help identify the problem.

## Discussion

In the sections above, we surveyed different types of neural networks and their applications to parameter estimation and model comparison. First, we showed that neural networks provide similar inferences and parameter recovery to traditional likelihood-based parameter estimation. Second, we showed that neural network classifiers substantially outperformed likelihood-based metrics for model comparison. By treating model comparison as a classification problem and training it on data simulated from each different theory or model, neural networks achieve much better performance than by comparing models using approximate metrics like BIC, AIC, WAIC, or other indices computed from the MCMC sampler during Bayesian inference. The neural network classifiers outperformed traditional Bayesian and classical approaches to identifying the true generative model by some distance. This was true for both the 27-item MCQ as well as other paradigms with varying stimuli and experiment lengths.

Third, we extended the capabilities of the neural networks we introduced initially to encompass flexible experimental paradigms and to quantify uncertainty in the form of a full Bayesian posterior. By incorporating an LSTM or other type of recurrent layer into the structure of a neural network ( Lueckmann et al., 2017; Rmus et al., 2023), it can handle sequential inputs – allowing it to fit time-variant models and to capture sequences of behavior that vary in length. This permitted the neural networks to handle intertemporal choice paradigms that varied from a handful to several hundred trials long while maintaining performance comparable (or in the case of model comparison, superior) to state-of-the-art Bayesian MCMC methods.

Finally, we showed that dropout layers can be added to a neural network to both reduce overfitting and vary its outputs from run to run. This allowed them to approximate a complete joint Bayesian posterior (Gal & Ghahramani, 2016) and do so several orders of magnitude faster than an MCMC sampler. The resulting posteriors from the neural network were comparable to those of the hierarchical Bayesian approach, suggesting no drop in descriptive accuracy or completeness.

### Parameter recovery

One advantage of the deep learning approach is that a modeler does not need to specify which characteristics of the data are most informative for estimating model parameters. In principle, a deep neural network with enough hidden layers or enough nodes in the hidden layers should be able to parse all of the information available from the behavioral data. As such, this work also doubles as an investigation into the recoverability of (some) models of intertemporal choice. Recent work has indicated that for many models, it can be difficult for an estimator to converge on the parameter values that were used to generate the data in the first place (Ballard et al., 2023).

A lack of recovery can speak as much to the method of parameter estimation as it can to the particular model used. Some estimators have trouble with noisy or multimodal likelihood functions (Oliver, 2017), correlations or trade-offs among parameters (Turner et al., 2013), or difficulties finding high-likelihood areas from their start points (Tanese, 1989; Gilks & Roberts, 1996). Removing the likelihood altogether using machine learning goes some distance toward alleviating these problems, although a poor choice of training set can still cause some problems (Sokratous et al., 2023). As we showed above, the joint posterior obtained from dropout sampling (Gal & Ghahramani, 2016) can help identify problematic parameters and identify simplifications that can reduce or eliminate estimation problems.

What we have shown here is that multiple different methods – hierarchical Bayesian/MCMC and likelihood-free machine learning – tend to agree on which parameters and models are difficult to recover. In particular, the time sensitivity parameters of both the hyperboloid and direct difference models are particularly difficult to accurately estimate. This suggests that they are poor candidates for endophenotypes of addiction, mental health, or other important real-world outcomes (Iacono, 2018), as any measured values are likely contaminated with error. Assessing time sensitivity may require different experimental paradigms, either eliciting different responses like prices or matching values (Kvam et al., 2021; Hardisty et al., 2013) or using entirely different approaches to understanding time perception, like duration discrimination, timing, or tapping tasks (Wing & Kristofferson, 1973; Brown, 2008; Ivry & Schlerf, 2008; Grondin, 2010).

On the other hand, our analyses suggest that discounting rates and attention weights are quite easy to recover, and can be estimated based on a minimal amount of data. Both hierarchical Bayesian and neural network methods agreed on the values of discounting rates and attention weights, and they correlated well with the true values whether we were using likelihood-based methods, fixed inputs, or the flexible-input grid method. As a result, it appears that discounting is readily assessable, and reinforces the idea that it can serve as a transdiagnostic metric for understanding many mental health, addiction, and real-world outcomes (Amlung et al., 2019; Levin et al., 2018). Its application to the real data on addiction above provides hints that it can be used to better understand impulsivity and its consequences, although it is perhaps not the strongest illustration to date (Kirby et al., 1999a; Bickel & Marsch, 2001; Bickel et al., 2008).

A stronger take-away here is that this understanding of impulsivity and addiction would not have been possible without one of the approaches to parameter estimation and model comparison. We strongly suggest that researchers focus on model-based methods for assessing discounting, as summary statistics of behavior alone are often not enough to accurately gauge the subjective value associated with delays (Haines et al., 2025; Bailey et al., 2021). The tools we have provided for doing so should result in better research and assessment related to impulsivity, mental health, and addiction.

### Which approach to use?

A major concern for many potential users has been that neural networks did not produce a complete posterior distribution over parameter values. Previous work had instead focused on training the network, or a secondary network, to approximate the error in its own estimates (e.g., Radev et al., 2020; Sokratous et al., 2023). This produced a posterior variance but not a joint distribution – meaning it missed out on important details like covariance among parameters. If this were the only method, a modeler would have had to instead approximate the likelihood function and then the posterior using more traditional posterior sampling methods like MCMC Fengler et al. (2021). Fortunately, simulation-based inference tools have been fully Bayesian for some time now (Lueckmann et al., 2021; Cranmer et al., 2020; Bürkner et al., 2023) dropout layers provide an efficient and accurate method for generating posterior distributions directly from the same neural network used to estimate best-fitting parameters.

Because the neural network is approximating a Gaussian process, its individual-level posteriors may not be fully robust to situations where the “true” posterior is clearly non-normal. To an extent, the modeler can control this by transforming the target parameters to unbounded scales – using log, logit, probit, square-root, or other functions to map them to continuous unbounded scales where they can be expected to approximate normality. However, in cases where the posterior shows sharp deviations from Gaussian distributions, it may be best to use likelihood approximations instead of direct posterior approximations (Fengler et al., 2021), or to use approaches like Bayesian neural networks that can draw posterior samples in such a way that they are not constrained to follow a Gaussian process (Izmailov et al., 2021).

The main advantage that hierarchical Bayesian methods have over neural networks is in cases where there are many conditions or manipulations. The entire data set, or some summary thereof, is required in order to form the inputs to the neural network. Conversely, hierarchical Bayesian methods can take any variety of conditions, stimuli, and responses and relatively easily adapt to increases or decreases in the number of conditions (and thus parameters) present in different data sets by iterating over these manipulations for specific parameters. For neural networks, the number of parameters is set by the number of outputs – meaning that a modeler must specify exactly what parameters change and how they change across conditions when constructing the inputs and outputs. A neural network will often need to be retrained for models with different parameters for different conditions because it has a fixed set of outputs – and thus is limited to estimating a fixed set of parameters at the same time. Therefore, it is hard to adapt them to situations where different participants saw different combinations of conditions (such as split-plot designs).

Of course, both approaches have to specify a parametric structure. The advantage of more traditional MCMC methods is that they can iterate over conditions using for loops, allowing the data to guide the number of conditions and number of parameters that must be estimated. By contrast, neural networks need to know in advance how many conditions and parameters to estimate. Future work on neural networks might consider flexible-output designs, where some outputs are unused for certain sets of inputs. In such an approach, the condition information could be supplied as a channel of the inputs, allowing the network to identify which output the data should inform. If successful, they might overcome this current barrier to modeling complex/variable experimental designs.

An additional note is that the neural network tends to be much more efficient than the likelihood-based approaches in terms of overall fitting time, as illustrated in Table 1. This difference only grows as the number of participants and parameters increases, meaning that more complex model and larger data sets will benefit even further than the rather simple models we used here.

Put together, the neural network provides posterior distributions of parameter estimates that are comparable to hierarchical Bayesian approaches, and does so much faster than MCMC methods. This suggests that it is particularly suitable for very large data sets, as it can carry out parameter estimation for thousands or even millions of participants in a short time (von Krause et al., 2022).

Our findings strongly suggest that neural networks can provide superior model comparison via classification, and so should be favored for these kinds of applications. We suspect that the superior performance of the networks as a model comparison technique follows from three main factors: (1) picking up on slight differences in data patterns that do not carry a large difference in associated log likelihood, despite being highly diagnostic of generative model; (b) doing a good job at integrating information about the frequency with which each model makes a particular prediction and thus incorporating model flexibility, and (c) the relative lack of information available from the log-likelihood by itself. Some of the interpretation of the best model depends on whether one assumes the “true” model is in the consideration set (Lee, 2018; Devezer et al., 2020; Lindley, 2013), as the true model is guaranteed to be in the training set but unlikely to be in the test set. That is, we know that all the data used to train the network came from a specific model, but we don’t know that the true data from participants actually came from any of the models we are considering. Detecting model mis-specification is another purview of neural networks that can begin to address this problem (Schmitt et al., 2023), although philosophically it is a much larger issue than we can do justice to here.

Naturally, neural networks should also be used when analytic likelihoods are not available, as MCMC methods typically require tractable likelihoods to be efficiently estimated. Conversely, Bayesian or other likelihood-based methods might be preferable when developing model code for many different experiments (where the number of parameters and conditions is unknown to the modeler in advance), especially those with complex experimental designs where many different parameters are needed for different conditions or situations where different participants see different combinations of stimuli. More traditional Bayesian approaches like MCMC might also be used when the posteriors are non-Gaussian – although it is critical to note that the likelihoods and priors used may impose assumptions of their own on the shape of the posteriors. There are also recent neural network-based methods for approximating non-Gaussian posteriors. These approaches are still relatively new (Papamakarios et al., 2021; Lenzi et al., 2023) but offer a promising new way to extend the methods we introduced here.

### New advances

The field of machine learning, and especially amortized Bayesian inference, is advancing at such a rate that there are likely to be meaningful advances even between when this paper is submitted and published. Already there are improved methods for handling data sets that vary in size and stimuli, such as transformers with context and attention layers (Vaswani et al., 2017), normalizing flows and other methods for obtaining exact Bayesian posteriors (Sainsbury-Dale et al., 2024; Cranmer et al., 2020; Radev et al., 2020; Gonçalves et al., 2020), and encoding different types of data more efficiently (Pinaya et al., 2020). Existing frameworks like BayesFlow (Radev et al., 2020) as well as more standard packages and programs like TensorFlow, PyTorch, and MATLAB Deep Learning Toolbox are making deep learning for parameter estimation and model comparison easier and more efficient every day. These approaches are now progressing much faster than advances in MCMC sampling, indicating that the difference in performance we showed here (e.g., Tables 1–2) will only grow over time. For those modelers considering a viable alternative to MCMC sampling with improved efficiency and accuracy, we recommend learning and adopting these machine learning approaches as well as keeping up with new innovations as they will undoubtedly unfold.

### Conclusion

In this paper, we examined the potential of neural networks for fitting the parameters of intertemporal choice models. Not only are these neural networks – once trained – much faster than traditional approaches to parameter estimation, but they can fit models that lack likelihood functions by relying purely on simulated data. Here, we have taken this work a step further to show that the estimates from neural network are on par with the optimized inference provided by cutting-edge methods like hierarchical Bayesian estimation: they produce estimates and posteriors comparable to Markov chain Monte Carlo methods. Furthermore, machine learning classifiers substantially exceed the capabilities of other methods when it comes to model comparison. Because of their speed and modular nature, trained neural networks can also be embedded in simple point-and-click tools (Kvam et al., 2024). We hope that the methods we have provided here form the basis for new modeling innovations beyond intertemporal choice, and provide a valid long-term alternative to likelihood-based methods of model fitting and comparison.

## Supplementary Information

Below is the link to the electronic supplementary material.

Supplementary file 1 (pdf 861 KB)

## Data Availability

All analysis and modeling were carried out in MATLAB 2023b using the Deep Learning Toolbox. Modeling and other analysis code are available on the Open Science Framework at osf.io/y4z82. Because the human data presented here are secondary, they are not freely available online. Instead, simulated data are provided on the OSF page that mimic the structure of the real data, which should allow readers to run the scripts provided and explore the output. For access to the real data set, please contact the last author. This study was not preregistered.

## Footnotes

1 We actually originally trained the networks on 10,000 simulated data sets. The smaller simulated data set size did not result in a huge difference relative to the 100,000 set size, but the networks tended to very slightly overfit in these cases, such that the loss for the training set (e.g., 0.37 for the direct difference model) was below that of the validation data set (e.g., 0.41 for the direct difference model) at the end of training.

## References

[CR1] Ahn, W. Y., Dai, J., Vassileva, J., Busemeyer, J. R., & Stout, J. C. (2016). Computational modeling for addiction medicine: From cognitive models to clinical applications. In *Progress in brain research* (Vol. 224, pp. 53–65). Elsevier. 10.1016/bs.pbr.2015.07.032 26822353

[CR2] Ahn, W.-Y., Vasilev, G., Lee, S.-H., Busemeyer, J. R., Kruschke, J. K., Bechara, A., & Vassileva, J. (2014). Decision-making in stimulant and opiate addicts in protracted abstinence: Evidence from computational modeling with pure users. *Frontiers in Psychology,**5*, 849. 25161631 10.3389/fpsyg.2014.00849 PMC4129374

[CR3] Ahn, W.-Y., & Vassileva, J. (2016). Machine-learning identifies substance-specific behavioral markers for opiate and stimulant dependence. *Drug and Alcohol Dependence,**161*, 247–257. 26905209 10.1016/j.drugalcdep.2016.02.008 PMC4955649

[CR4] Ainslie, G. W., & Haslam, N. (1992). *Hyperbolic discounting*. Russell Sage Foundation.

[CR5] Akaike, H. (1998). Information theory and an extension of the maximum likelihood principle. In *Selected papers of Hirotugu Akaike* (pp. 199–213). Springer.

[CR6] Amasino, D. R., Sullivan, N. J., Kranton, R. E., & Huettel, S. A. (2019). Amount and time exert independent influences on intertemporal choice. *Nature Human Behaviour,**3*(4), 383–392. 30971787 10.1038/s41562-019-0537-2 PMC8020819

[CR7] Amlung, M., Marsden, E., Holshausen, K., Morris, V., Patel, H., Vedelago, L., Naish, K. R., Reed, D. D., & McCabe, R. E. (2019). Delay discounting as a transdiagnostic process in psychiatric disorders: a meta-analysis. *JAMA Psychiatry,**76*(11), 1176–1186. 31461131 10.1001/jamapsychiatry.2019.2102 PMC6714026

[CR8] Bailey, A. J., Romeu, R. J., & Finn, P. R. (2021). The problems with delay discounting: A critical review of current practices and clinical applications. *Psychological Medicine,**51*(11), 1799–1806. 34184631 10.1017/S0033291721002282 PMC8381235

[CR9] Baldi, P., & Sadowski, P. J. (2013). Understanding dropout. *Advances in Neural Information Processing Systems*, *26*.

[CR10] Ballard, T., Luckman, A., & Konstantinidis, E. (2023). A systematic investigation into the reliability of inter-temporal choice model parameters. *Psychonomic Bulletin & Review,**30*(4), 1294–1322. 36877362 10.3758/s13423-022-02241-7 PMC10482820

[CR11] Bickel, W. K., Jarmolowicz, D. P., Mueller, E. T., Koffarnus, M. N., & Gatchalian, K. M. (2012). Excessive discounting of delayed reinforcers as a trans-disease process contributing to addiction and other disease-related vulnerabilities: Emerging evidence. *Pharmacology & therapeutics,**134*(3), 287–297. 22387232 10.1016/j.pharmthera.2012.02.004 PMC3329584

[CR12] Bickel, W. K., Johnson, M. W., Koffarnus, M. N., MacKillop, J., & Murphy, J. G. (2014). The behavioral economics of substance use disorders: Reinforcement pathologies and their repair. *Annual Review of Clinical Psychology,**10*, 641–677. 24679180 10.1146/annurev-clinpsy-032813-153724 PMC4501268

[CR13] Bickel, W. K., & Marsch, L. A. (2001). Toward a behavioral economic understanding of drug dependence: Delay discounting processes. *Addiction,**96*(1), 73–86. 11177521 10.1046/j.1360-0443.2001.961736.x

[CR14] Bickel, W. K., Yi, R., Kowal, B. P., & Gatchalian, K. M. (2008). Cigarette smokers discount past and future rewards symmetrically and more than controls: Is discounting a measure of impulsivity? *Drug and Alcohol Dependence,**96*(3), 256–262. 18468814 10.1016/j.drugalcdep.2008.03.009 PMC2701143

[CR15] Bickel, W. K., Yi, R., Landes, R. D., Hill, P. F., & Baxter, C. (2011). Remember the future: Working memory training decreases delay discounting among stimulant addicts. *Biological Psychiatry,**69*(3), 260–265. 20965498 10.1016/j.biopsych.2010.08.017 PMC3015021

[CR16] Bonato, M., Zorzi, M., & Umiltá, C. (2012). When time is space: evidence for a mental time line. *Neuroscience & Biobehavioral Reviews,**36*(10), 2257–2273. 22935777 10.1016/j.neubiorev.2012.08.007

[CR17] Brown, S. W. (2008). *Time and Attention: Review of the Literature*. In Psychology of time: Emerald Group Publishing.

[CR18] Bürkner, P.-C., Scholz, M., & Radev, S. T. (2023). Some models are useful, but how do we know which ones? towards a unified Bayesian model taxonomy. *Statistic Surveys,**17*, 216–310.

[CR19] Busemeyer, J. R., & Stout, J. C. (2002). A contribution of cognitive decision models to clinical assessment: Decomposing performance on the Bechara gambling task. *Psychological Assessment,**14*(3), 253. 12214432 10.1037//1040-3590.14.3.253

[CR20] Cavagnaro, D. R., Aranovich, G. J., McClure, S. M., Pitt, M. A., & Myung, J. I. (2016). On the functional form of temporal discounting: An optimized adaptive test. *Journal of Risk and Uncertainty,**52*, 233–254. 29332995 10.1007/s11166-016-9242-y PMC5764197

[CR21] Cavagnaro, D. R., Myung, J. I., Pitt, M. A., & Kujala, J. V. (2010). Adaptive design optimization: A mutual information-based approach to model discrimination in cognitive science. *Neural Computation,**22*(4), 887–905. 20028226 10.1162/neco.2009.02-09-959

[CR22] Chen, X., & Zhao, X. (2024). How time flies: Time perception and intertemporal choice. *Journal of Behavioral and Experimental Economics,**109*, Article 102160.

[CR23] Cheng, J., & González-Vallejo, C. (2016). Attribute-wise vs. alternative-wise mechanism in intertemporal choice: Testing the proportional difference, trade-off, and hyperbolic models. *Decision,**3*(3), 190.

[CR24] Conrod, P. J., Castellanos-Ryan, N., & Strang, J. (2010). Brief, personality-targeted coping skills interventions and survival as a non–drug user over a 2-year period during adolescence. *Archives of General Psychiatry,**67*(1), 85–93. 20048226 10.1001/archgenpsychiatry.2009.173

[CR25] Cranmer, K., Brehmer, J., & Louppe, G. (2020). The frontier of simulation-based inference. *Proceedings of the National Academy of Sciences,**117*(48), 30055–30062. 10.1073/pnas.1912789117 PMC7720103 32471948

[CR26] Dai, J., & Busemeyer, J. R. (2014). A probabilistic, dynamic, and attribute-wise model of intertemporal choice. *Journal of Experimental Psychology: General,**143*(4), 1489. 24635188 10.1037/a0035976 PMC4115005

[CR27] Dai, J., Gunn, R. L., Gerst, K. R., Busemeyer, J. R., & Finn, P. R. (2016). A random utility model of delay discounting and its application to people with externalizing psychopathology. *Psychological Assessment,**28*(10), 1198. 26595217 10.1037/pas0000248 PMC4877297

[CR28] Dai, J., Pleskac, T. J., & Pachur, T. (2018). Dynamic cognitive models of intertemporal choice. *Cognitive Psychology,**104*, 29–56. 29587183 10.1016/j.cogpsych.2018.03.001

[CR29] Devezer, B., Navarro, D. J., Vandekerckhove, J., & Ozge Buzbas, E. (2020). The case for formal methodology in scientific reform. *Royal Society open science,**8*(3), Article 200805. 10.1098/rsos.200805 PMC8101540 34035933

[CR30] Donohew, L., Zimmerman, R., Cupp, P. S., Novak, S., Colon, S., & Abell, R. (2000). Sensation seeking, impulsive decision-making, and risky sex: Implications for risk-taking and design of interventions. *Personality and Individual Differences,**28*(6), 1079–1091.

[CR31] Elsemüller, L., Schnuerch, M., Bürkner, P.-C., & Radev, S. T. (2023). A deep learning method for comparing Bayesian hierarchical models. arXiv preprint arXiv:2301.11873. 10.1037/met0000645 38709626

[CR32] Elsemüller, L., Schnuerch, M., Bürkner, P.-C., & Radev, S. T. (2024). A deep learning method for comparing Bayesian hierarchical models. *Psychological Methods*. 10.1037/met0000645 38709626

[CR33] Fengler, A., Govindarajan, L. N., Chen, T., & Frank, M. J. (2021). Likelihood approximation networks (lans) for fast inference of simulation models in cognitive neuroscience. *Elife,**10*, Article e65074. 33821788 10.7554/eLife.65074 PMC8102064

[CR34] Fengler, A., Govindarajan, L. N., & Frank, M. J. (2020). Encoder-decoder neural architectures for fast amortized inference of cognitive process models. In S. Denison, M. Mack, Y. Xu, & B. C. Armstrong (Eds.), . cognitivesciencesociety.org.

[CR35] Franco-Watkins, A. M., Mattson, R. E., & Jackson, M. D. (2016). Now or later? attentional processing and intertemporal choice. *Journal of Behavioral Decision Making,**29*(2–3), 206–217.

[CR36] Gal, Y., & Ghahramani, Z. (2016). Dropout as a Bayesian approximation: Representing model uncertainty in deep learning. In international conference on machine learning (pp. 1050–1059).

[CR37] Gelfand, A. E. (2000). Gibbs sampling. *Journal of the American Statistical Association,**95*(452), 1300–1304.

[CR38] Gilks, W. R., & Roberts, G. O. (1996). Strategies for improving mcmc. *Markov chain Monte Carlo in practice,**6*, 89–114.

[CR39] Gonçalves, P. J., Lueckmann, J.-M., Deistler, M., Nonnenmacher, M., Öcal, K., Bassetto, G., Chintaluri, C., Podlaski, W.F., Haddad, S.A., Vogels, T.P., & Greenberg, D.S. (2020). Training deep neural density estimators to identify mechanistic models of neural dynamics. *elife*, *9*, e56261. 10.7554/eLife.56261 PMC7581433 32940606

[CR40] Green, L., & Myerson, J. (1996). Exponential versus hyperbolic discounting of delayed outcomes: Risk and waiting time. *American Zoologist,**36*(4), 496–505.

[CR41] Grondin, S. (2010). Timing and time perception: A review of recent behavioral and neuroscience findings and theoretical directions. *Attention, Perception, & Psychophysics,**72*(3), 561–582. 10.3758/APP.72.3.561 20348562

[CR42] Guo, C., Pleiss, G., Sun, Y., & Weinberger, K. Q. (2017). On calibration of modern neural networks. In *International conference on machine learning* (pp. 1321–1330).

[CR43] Haines, N., Kvam, P. D., Irving, L. H., Smith, C., Beauchaine, T. P., Pitt, M. A., Ahn, W.Y., & Turner, B. (2025). Learning from the reliability paradox: How theoretically informed generative models can advance the social, behavioral, and brain sciences. *Psychological Methods*. 10.1037/met0000674 40232753

[CR44] Haines, N., Sullivan-Toole, H., & Olino, T. (2023). From classical methods to generative models: Tackling the unreliability of neuroscientific measures in mental health research. *Biological Psychiatry: Cognitive Neuroscience and Neuroimaging.,**8*(8), 822–831. 36997406 10.1016/j.bpsc.2023.01.001 PMC10333448

[CR45] Hardisty, D. J., Thompson, K. F., Krantz, D. H., & Weber, E. U. (2013). How to measure time preferences: An experimental comparison of three methods. *Judgment and Decision making,**8*(3), 236–249.

[CR46] Hedge, C., Powell, G., & Sumner, P. (2018). The reliability paradox: Why robust cognitive tasks do not produce reliable individual differences. *Behavior Research Methods,**50*(3), 1166–1186. 28726177 10.3758/s13428-017-0935-1 PMC5990556

[CR47] Hoffman, M. D., Gelman, A., et al. (2014). The no-u-turn sampler: adaptively setting path lengths in hamiltonian monte carlo. *Journal of Machine Learning Research,**15*(1), 1593–1623.

[CR48] Iacono, W. G. (2018). Endophenotypes in psychiatric disease: prospects and challenges. *Genome medicine,**10*, 1–3. 29471866 10.1186/s13073-018-0526-5 PMC5824588

[CR49] Ivry, R. B., & Schlerf, J. E. (2008). Dedicated and intrinsic models of time perception. *Trends in Cognitive Sciences,**12*(7), 273–280. 18539519 10.1016/j.tics.2008.04.002 PMC4335014

[CR50] Izmailov, P., Vikram, S., Hoffman, M. D., & Wilson, A. G. G. (2021). What are Bayesian neural network posteriors really like? In *International conference on machine learning* (pp. 4629–4640).

[CR51] Kaplan, B. A., Amlung, M., Reed, D. D., Jarmolowicz, D. P., McKerchar, T. L., & Lemley, S. M. (2016). Automating scoring of delay discounting for the 21-and 27-item monetary choice questionnaires. *The Behavior Analyst,**39*, 293–304. 31976983 10.1007/s40614-016-0070-9 PMC6701266

[CR52] Kapoor, J., Schulz, A., Vetter, J., Pei, F., Gao, R., & Macke, J. H. (2024). Latent diffusion for neural spiking data. *Advances in Neural Information Processing Systems,**37*, 118119–118154.

[CR53] Kass, R. E., & Raftery, A. E. (1995). *Bayes factor.,**90*(430), 773–795. 10.2307/2291091

[CR54] Killeen, P. R., & Grondin, S. (2021). A trace theory of time perception. *Psychological Review*. 10.1037/rev0000308 34553968

[CR55] Kingma, D. P., & Ba, J. (2014). *Adam: A method for stochastic optimization*.

[CR56] Kirby, K. N., Petry, N. M., & Bickel, W. K. (1999). Heroin addicts have higher discount rates for delayed rewards than non-drug-using controls. *Journal of Experimental Psychology: General,**128*(1), 78. 10100392 10.1037//0096-3445.128.1.78

[CR57] Kirby, K. N., Petry, N. M., & Bickel, W. K. (1999b). Monetary choice questionnaire. *Journal of Experimental Psychology: General*. 10.1037//0096-3445.128.1.78 10100392

[CR58] Kruschke, J. K. (2014). *Doing Bayesian data analysis: A tutorial with R, JAGS, and STAN*. Academic Press.

[CR59] Kruschke, J. K. (2021). Bayesian analysis reporting guidelines. *Nature Human Behaviour,**5*(10), 1282–1291. 34400814 10.1038/s41562-021-01177-7 PMC8526359

[CR60] Kvam, P. D., Baldwin, M., & Westgate, E. C. (2023). Cognitive mechanisms underlying subjective value of past and future events: Modeling systematic reversals of temporal value asymmetry. *Decision,**10*, 1–30.

[CR61] Kvam, P. D., Irving, L. H., Sokratous, K., & Smith, C. T. (2024). Improving the reliability and validity of the iat with a dynamic model driven by similarity. *Behavior Research Methods,**56*(3), 2158–2193. 37450219 10.3758/s13428-023-02141-1

[CR62] Kvam, P. D., Romeu, R. J., Turner, B. M., Vassileva, J., & Busemeyer, J. R. (2021). Testing the factor structure underlying behavior using joint cognitive models: Impulsivity in delay discounting and cambridge gambling tasks. *Psychological Methods,**26*(1), 18–37. 32134313 10.1037/met0000264 PMC7483167

[CR63] Kvam, P. D., Sokratous, K., & Fitch, A. (2025). Decisions among shifting choice alternatives reveal option-general representations of evidence. *Psychological Review,**132*(3), 528–555. 39298219 10.1037/rev0000500

[CR64] Kvam, P. D., Sokratous, K., Fitch, A., & Hintze, A. (in press). Using artificial intelligence to fit, compare, evaluate, and discover computational models of decision behavior. *Decision*.

[CR65] Kvam, P. D., Sokratous, K., Johnson, G., Lin, S. T., & Unruh, E. (2021). Preference reversals between intertemporal choice and pricing. In *Proceedings of the annual meeting of the cognitive science society* (Vol. 43).

[CR66] Lee, D. G., & Hare, T. A. (2023). Evidence accumulates for individual attributes during value-based decisions. *Decision,**10*(4), 330–346.

[CR67] Lee, M. D. (2018). Bayesian methods in cognitive modeling. *The Stevens’ Handbook of Experimental Psychology and Cognitive Neuroscience,**5*, 37–84.

[CR68] Lejuez, C. W., Aklin, W. M., Jones, H. A., Richards, J. B., Strong, D. R., Kahler, C. W., & Read, J. P. (2003). The balloon analogue risk task (BART) differentiates smokers and nonsmokers. *Experimental and Clinical Psychopharmacology,**11*(1), 26. 12622341 10.1037//1064-1297.11.1.26

[CR69] Lenzi, A., Bessac, J., Rudi, J., & Stein, M. L. (2023). Neural networks for parameter estimation in intractable models. *Computational Statistics & Data Analysis,**185*, Article 107762.

[CR70] Levin, M. E., Haeger, J., Ong, C. W., & Twohig, M. P. (2018). An examination of the transdiagnostic role of delay discounting in psychological inflexibility and mental health problems. *The Psychological Record,**68*, 201–210.

[CR71] Lindley, D. V. (2013). *Understanding uncertainty*. Hoboken, John Wiley & Sons.

[CR72] Loewenstein, G., & Thaler, R. H. (1989). Anomalies: intertemporal choice. *Journal of Economic perspectives,**3*(4), 181–193.

[CR73] Luce, R. D. (1959). *Individual choice behavior: A theoretical analysis*. Hoboken: John Wiley and Sons.

[CR74] Lueckmann, J.-M., Boelts, J., Greenberg, D., Goncalves, P., & Macke, J. (2021). Benchmarking simulation-based inference. In *International conference on artificial intelligence and statistics* (pp. 343–351).

[CR75] Lueckmann, J.-M., Goncalves, P. J., Bassetto, G., Öcal, K., Nonnenmacher, M., & Macke, J. H. (2017). Flexible statistical inference for mechanistic models of neural dynamics. *Advances in neural information processing systems*, *30*.

[CR76] MacKillop, J., Amlung, M. T., Few, L. R., Ray, L. A., Sweet, L. H., & Munafó, M. R. (2011). Delayed reward discounting and addictive behavior: A meta-analysis. *Psychopharmacology (Berl),**216*(3), 305–321. 21373791 10.1007/s00213-011-2229-0 PMC3201846

[CR77] Madden, G. J., Bickel, W. K., & Jacobs, E. A. (1999). Discounting of delayed rewards in opioid-dependent outpatients: exponential or hyperbolic discounting functions? *Experimental and Clinical Psychopharmacology,**7*(3), 284. 10472517 10.1037//1064-1297.7.3.284

[CR78] Madden, G. J., & Johnson, P. S. (2010). *A delay-discounting primer*. In Impulsivity: The behavioral and neurological science of discounting. American Psychological Association.

[CR79] Mazur, J. E. (1987). An adjusting procedure for studying delayed reinforcement. Commons, ML.; Mazur, JE.; Nevin, JA, 55–73.

[CR80] Mitchell, S. H. (2019). Linking delay discounting and substance use disorders: genotypes and phenotypes. *Perspectives on Behavior Science,**42*(3), 419–432. 31976442 10.1007/s40614-019-00218-x PMC6768927

[CR81] Molloy, M. F., Romeu, R. J., Kvam, P. D., Finn, P. R., Busemeyer, J., & Turner, B. M. (2020). Hierarchies improve individual assessment of temporal discounting behavior. *Decision,**7*, 212–224. 34621906 10.1037/dec0000121 PMC8493833

[CR82] Mortier, T., Bengs, V., Hüllermeier, E., Luca, S., & Waegeman, W. (2023). On the calibration of probabilistic classifier sets. In *International conference on artificial intelligence and statistics* (pp. 8857–8870).

[CR83] Myerson, J., & Green, L. (1995). Discounting of delayed rewards: Models of individual choice. *Journal of the Experimental Analysis of Behavior,**64*(3), 263–276. 16812772 10.1901/jeab.1995.64-263 PMC1350137

[CR84] Myerson, J., Green, L., & Warusawitharana, M. (2001). Area under the curve as a measure of discounting. *Journal of the Experimental Analysis of Behavior,**76*(2), 235–243. 11599641 10.1901/jeab.2001.76-235 PMC1284836

[CR85] Myung, I. J. (2003). Tutorial on maximum likelihood estimation. *Journal of Mathematical Psychology,**47*(1), 90–100. 10.1016/S0022-2496(02)00028-7

[CR86] Neal, R. M. (2003). *Slice sampling. The annals of statistics,**31*(3), 705–767.

[CR87] Neal, R. M., et al. (2011). Mcmc using hamiltonian dynamics. *Handbook of markov chain monte carlo,**2*(11), 2.

[CR88] Odum, A. L. (2011). Delay discounting: I’m ak, you’re ak. *Journal of the Experimental Analysis of Behavior,**96*(3), 427–439. 22084499 10.1901/jeab.2011.96-423 PMC3213005

[CR89] Oh, K.-S., & Jung, K. (2004). Gpu implementation of neural networks. *Pattern Recognition,**37*(6), 1311–1314.

[CR90] Oliver, D. S. (2017). Metropolized randomized maximum likelihood for improved sampling from multimodal distributions. *SIAM/ASA Journal on Uncertainty Quantification,**5*(1), 259–277.

[CR91] Papamakarios, G., Nalisnick, E., Rezende, D. J., Mohamed, S., & Lakshminarayanan, B. (2021). Normalizing flows for probabilistic modeling and inference. *Journal of Machine Learning Research,**22*(57), 1–64.

[CR92] Pinaya, W. H. L., Vieira, S., Garcia-Dias, R., & Mechelli, A. (2020). Autoencoders. In *Machine learning* (pp. 193–208). Elsevier.

[CR93] Rachlin, H. (2006). Notes on discounting. *Journal of the Experimental Analysis of Behavior,**85*(3), 425–435. 16776060 10.1901/jeab.2006.85-05 PMC1459845

[CR94] Rachlin, H., Logue, A. W., Gibbon, J., & Frankel, M. (1986). Cognition and behavior in studies of choice. *Psychological Review,**93*(1), 33.

[CR95] Radev, S. T., D’Alessandro, M., Mertens, U. K., Voss, A., Köthe, U., & Bürkner, P.-C. (2021). Amortized Bayesian model comparison with evidential deep learning. *IEEE Transactions on Neural Networks and Learning Systems,**34*(8), 4903–4917. 10.1109/TNNLS.2021.3124052 34767511

[CR96] Radev, S. T., Mertens, U. K., Voss, A., Ardizzone, L., & Köthe, U. (2020). *Bayesflow: Learning complex stochastic models with invertible neural networks*. 10.1109/TNNLS.2020.3042395 33338021

[CR97] Radev, S. T., Mertens, U. K., Voss, A., & Köthe, U. (2020). Towards end-to-end likelihood-free inference with convolutional neural networks. *British Journal of Mathematical and Statistical Psychology,**73*(1), 23–43. 30793299 10.1111/bmsp.12159

[CR98] Raftery, A. E. (1995). Bayesian model selection in social research. *Sociological Methodology*, 111–163.

[CR99] Reynolds, B. (2006). A review of delay-discounting research with humans: Relations to drug use and gambling. *Behavioural Pharmacology,**17*(8), 651–667. 17110792 10.1097/FBP.0b013e3280115f99

[CR100] Rmus, M., Pan, T.-F., Xia, L., & Collins, A. G. (2023). Artificial neural networks for model identification and parameter estimation in computational cognitive models. *Biorxiv*. 10.1371/journal.pcbi.1012119 PMC11132492 38748770

[CR101] Rodriguez, C. A., Turner, B. M., & McClure, S. M. (2014). Intertemporal choice as discounted value accumulation. *PLoS ONE,**9*(2), Article e90138. 24587243 10.1371/journal.pone.0090138 PMC3938649

[CR102] Romeu, R. J., Haines, N., Ahn, W.-Y., Busemeyer, J. R., & Vassileva, J. (2019). A computational model of the Cambridge gambling task with applications to substance use disorders. *Drug and Alcohol Dependence*, 107711. 10.1016/j.drugalcdep.2019.107711 PMC6980771 31735532

[CR103] Sainsbury-Dale, M., Zammit-Mangion, A., & Huser, R. (2024). Likelihood-free parameter estimation with neural bayes estimators. *The American Statistician,**78*(1), 1–14.

[CR104] Sainsbury-Dale, M., Zammit-Mangion, A., Richards, J., & Huser, R. (2025). Neural bayes estimators for irregular spatial data using graph neural networks. *Journal of Computational and Graphical Statistics*, 1–16.

[CR105] Sawicki, P., & Białek, M. (2016). Side effects in time discounting procedures: Fixed alternatives become the reference point. *PLoS ONE,**11*(10), Article e0165245. 27768759 10.1371/journal.pone.0165245 PMC5074525

[CR106] Schmitt, M., Bürkner, P.-C., Köthe, U., & Radev, S. T. (2023). Detecting model misspecification in amortized Bayesian inference with neural networks. In *Dagm german conference on pattern recognition* (pp. 541–557).

[CR107] Scholten, M., & Read, D. (2010). The psychology of intertemporal tradeoffs. *Psychological Review,**117*(3), 925. 20658858 10.1037/a0019619

[CR108] Scholten, M., Read, D., & Sanborn, A. (2014). Weighing outcomes by time or against time? evaluation rules in intertemporal choice. *Cognitive Science,**38*(3), 399–438. 24404941 10.1111/cogs.12104

[CR109] Schumacher, L., Bürkner, P.-C., Voss, A., Köthe, U., & Radev, S. T. (2023). Neural superstatistics for Bayesian estimation of dynamic cognitive models. *Scientific Reports,**13*(1), 13778. 37612320 10.1038/s41598-023-40278-3 PMC10447473

[CR110] Schwarz, G. (1978). Estimating the dimension of a model. *The Annals of Statistics,**6*(2), 461–464. 10.1214/aos/1176344136

[CR111] Shiffrin, R., Lee, M., Kim, W., & Wagenmakers, E.-J. (2008). A survey of model evaluation approaches with a tutorial on hierarchical Bayesian methods. *Cognitive Science: A Multidisciplinary Journal,**32*(8), 1248–1284. 10.1080/03640210802414826 10.1080/03640210802414826 21585453

[CR112] Sokratous, K., Fitch, A., & Kvam, P. D. (2023). How to ask twenty questions and win: Machine learning tools for assessing preferences from small samples of willingness-to-pay prices. *Journal of Choice Modelling,**48*, Article 100418.

[CR113] Spiegelhalter, D. J., Best, N. G., Carlin, B. P., & Linde, A. (2014). The deviance information criterion: 12 years on. *Journal of the Royal Statistical Society, Series B: Statistical Methodology,**76*(3), 485–493.

[CR114] Stevenson, M. K. (1986). A discounting model for decisions with delayed positive or negative outcomes. *Journal of Experimental Psychology: General,**115*(2), 131–154.

[CR115] Sullivan-Toole, H., Haines, N., Dale, K., & Olino, T. M. (2022). Enhancing the psychometric properties of the iowa gambling task using full generative modeling. *Computational Psychiatry,**6*(1), 189. 37332395 10.5334/cpsy.89 PMC10275579

[CR116] Tanese, R. (1989). *Distributed genetic algorithms for function optimization*. Ann Arbor: University of Michigan.

[CR117] Turner, B. M., Sederberg, P. B., Brown, S. D., & Steyvers, M. (2013). A method for efficiently sampling from distributions with correlated dimensions. *Psychological Methods,**18*(3), 368–384. 23646991 10.1037/a0032222 PMC4140408

[CR118] Vassileva, J., & Conrod, P. J. (2018). Impulsivities and addictions: A multidimensional integrative framework informing assessment and interventions for substance use disorders. *Philosophical Transactions of the Royal Society B,**374*(1766), 20180137. 10.1098/rstb.2018.0137 PMC6335463 30966920

[CR119] Vaswani, A., Shazeer, N., Parmar, N., Uszkoreit, J., Jones, L., Gomez, A. N., Kaiser, Ł., & Polosukhin, I. (2017). Attention is all you need. *Advances in Neural Information Processing Systems*, *30*.

[CR120] Vehtari, A., Gelman, A., & Gabry, J. (2017). Practical Bayesian model evaluation using leave-one-out cross-validation and waic. *Statistics and Computing,**27*, 1413–1432.

[CR121] Vehtari, A., Gelman, A., Simpson, D., Carpenter, B., & Bürkner, P.-C. (2021). Rank-normalization, folding, and localization: An improved r-hat for assessing convergence of MCMC (with discussion). *Bayesian Analysis,**16*(2), 667–718.

[CR122] Vincent, B. T., & Stewart, N. (2020). The case of muddled units in temporal discounting. *Cognition,**198*, Article 104203. 10.1016/j.cognition.2020.104203 32007801

[CR123] von Krause, M., Radev, S. T., & Voss, A. (2022). Mental speed is high until age 60 as revealed by analysis of over a million participants. *Nature Human Behaviour,**6*(5), 700–708. 10.1038/s41562-021-01282-7 35177809

[CR124] Wallsten, T. S., & Pleskac, T. J. (2005). Modeling behavior in a clinically diagnostic sequential risk-taking task. *Psychological Review,**112*(4), 862–880. 16262471 10.1037/0033-295X.112.4.862

[CR125] Watanabe, S., & Opper, M. (2010). Asymptotic equivalence of bayes cross validation and widely applicable information criterion in singular learning theory. *Journal of Machine Learning Research*, *11*(12).

[CR126] Wing, A. M., & Kristofferson, A. (1973). The timing of interresponse intervals. *Perception & Psychophysics,**13*(3), 455–460.

[CR127] Yechiam, E., Busemeyer, J. R., Stout, J. C., & Bechara, A. (2005). Using cognitive models to map relations between neuropsychological disorders and human decision-making deficits. *Psychological Science,**16*(12), 973–978. 16313662 10.1111/j.1467-9280.2005.01646.x

[CR128] Yi, R., Gatchalian, K. M., & Bickel, W. K. (2006). Discounting of past outcomes. *Experimental and Clinical Psychopharmacology,**14*(3), 311–317. 16893274 10.1037/1064-1297.14.3.311

[CR129] Zammit-Mangion, A., Sainsbury-Dale, M., & Huser, R. (2024). Neural methods for amortised parameter inference. *arXiv e-prints*, arXiv–2404.

[CR130] Zilker, V. (2022). Choice rules can affect the informativeness of model comparisons. *Computational Brain & Behavior,**5*(3), 397–421.

[CR131] Zois, E., Kortlang, N., Vollstädt-Klein, S., Lemenager, T., Beutel, M., Mann, K., & Fauth-Bühler, M. (2014). Decision-making deficits in patients diagnosed with disordered gambling using the Cambridge Gambling task: The effects of substance use disorder comorbidity. *Brain and behavior,**4*(4), 484–494. 25161815 10.1002/brb3.231 PMC4107466

