# Supplementary material for: Comparing likelihood-based and likelihood-free approaches to fitting and comparing models of intertemporal choice
Source: Behav Res Methods. 2025 Aug 11;57(9):252. doi: 10.3758/s13428-025-02779-z (PMC12339600; doi:10.3758/s13428-025-02779-z)
Supplement: Supplementary file 1 — (pdf 861 KB) [file 13428_2025_2779_MOESM1_ESM.pdf]

Supplementary materials for "Comparing likelihood-based and likelihood-free approaches to fitting and comparing models of intertemporal choice"

Peter D. Kvam[1], Konstantina Sokratous[2], Anderson K. Fitch[2], and Jasmin Vassileva[3]

[1]The Ohio State University

[2]University of Florida

[3]Virginia Commonwealth University

Supplementary materials for "Comparing likelihood-based and likelihood-free approaches to fitting and comparing models of intertemporal choice"

Gaussian process grid

Aside from LSTMs or other recurrent layers, a second way to address the flexibility problem is to use a generalized input structure that applies to data with any number of trials, combinations of stimuli, and degree of certainty about responses. The inputs in this case should as closely as possible describe the complete data set, losing as little information as possible while summarizing the stimuli and responses with a consistent set of values that characterize performance on the task. To meet this criteria, we created a multidimensional Gaussian process that characterizes performance on the task as a relationship between sets of four predictors (attributes of SS and LL options) and decisions that simulated participants made.

The simulated data we used were the same as those described above in the LSTM section. To create the network input, we first transformed the (simulated) behavioral data into a set of 5-dimensional points, where the first dimension was the payoff of the smaller-soon option, the second dimension was the payoff of the larger-later option, the third dimension was the delay to receipt for the smaller-sooner option, the fourth dimension was the delay to receipt for the larger-later option, and the final dimension was a participant's response. A set of data, therefore, consists of any number of 5-dimensional points, where the first four dimensions are predictors (stimuli) and the last dimension is the behavioral outcome (choice; 1 = larger-later, 0 = smaller-sooner).

The sets of 4 predictors and 1 outcome were then fed into a Gaussian process (GP) regression (fitrgp in MATLAB), using a squared exponential kernel, uncertainty (i.e., assuming non-deterministic outcomes), and default optimized settings for the GP kernel. Once this was done, we calculated the mean prediction and degree of uncertainty of the GP at each of 10,000 points (10 SS payoffs $\times$ 10 LL payoffs $\times$ 10 SS delays $\times$ 10 LL delays) arranged in a grid. This produced a set of 20,000 inputs (10,000 means and 10,000 error estimates) which could be used as an input to the neural network.

Specifically, we took values of \$5,\$15, ... \$95 for the smaller-sooner option (*SS*), values of *SS*+\$5, *SS*+\$15, ... , *SS*+\$95 for the larger later option (*LL*), delays of 0 days, 20 days, ..., 180 days for the smaller sooner option (t_{SS}), and delays of t_{SS}+5 days, t_{SS}+25 days, ..., t_{SS}+185 days for the larger later option (t_{LL}). This allowed us to accommodate a reasonable range of stimuli that a participant might see during a typical intertemporal choice experiment, ranging from \$5-180 and 0-365 days. At each combination of these ($10 \times 10 \times 10 \times 10 = 10{,}000$) points in the grid, we pulled the mean estimate of the probability of selecting the LL option as well as the standard deviation from the GP estimated from the data. In other words, we obtained a grid of points mapping stimuli onto response probabilities alongside error terms, providing a relatively complete description of behavior and uncertainty across the stimuli that simulated participants could encounter.

For larger data sets with more points informing the GP, the error terms will naturally be smaller, whereas smaller data sets with fewer combinations of stimuli will of course result in higher error estimates. The size of a data set is therefore “baked in” to the grid input, without having to vary the actual size of the input itself. This therefore lets us model data consisting of very many or very few stimuli using the exact same method to turn raw data into network inputs.

Results

As with the fixed-input and LSTM methods covered above, we are most interested in benchmarking the neural network against the best model-fitting method available, which was the hierarchical Bayesian method of fitting each of the models (Molloy et al., 2020). As before, we examine the ability of each method to recover the true parameter values that were used to generate a set of simulated data. We also tested the trained models and hierarchical Bayesian methods on an out-of-sample validation set to avoid making conclusions based on over-fit networks.

The performance of the neural network and Bayesian approaches are shown in Figure S1; readers will note that the hierarchical Bayesian half of the scatterplots (orange) are identical to those shown in main text Figure 2. As with the LSTM method, the neural network with grid input performed slightly better in recovering discounting rates for both the hyperbolic and hyperboloid

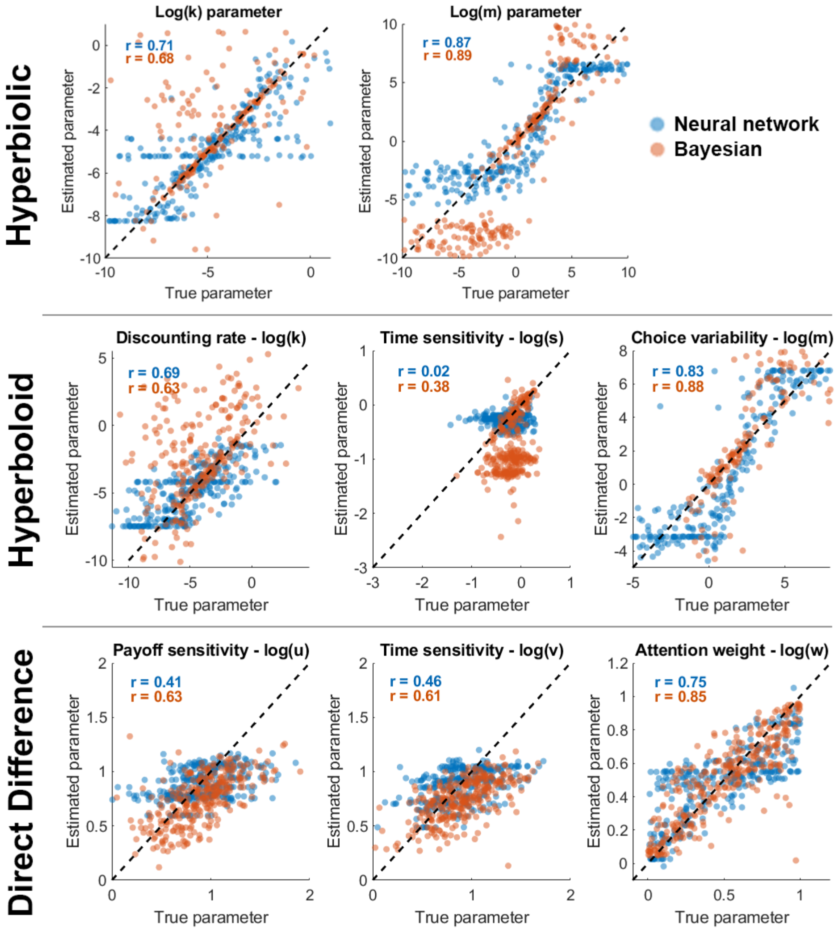

Figure S1

Relationship between true (x) and estimated (y) parameter values for estimates generated by the simulation inversion networks (blue) and hierarchical Bayesian implementations of the models (orange) for each of the three models, using the grid-based input from the Gaussian process.

models, slightly worse for the time sensitivity parameter, and the same as the Bayesian method for all other parameters.

In addition to the tests carried out in the main text, there are a few checks to carry out that should help establish confidence in the findings presented in the main paper. The first of these is to check the performance of the MCMC sampler, and the second is to evaluate how the “prior” – in the case of the neural network, the training set – affects parameter estimates and conclusions that we can draw from the model. We present the outcomes of analyses checking both of these below.

Sampler properties

The first check to carry out is to check that the MCMC sampler we used is behaving properly. If the results are inconsistent, or the sampler is not exploring the parameter space effectively, then that could limit its overall performance in both parameter estimation and model comparison.

To evaluate convergence, we both examined the traceplots for the MCMC chains and examine the r-hat values. Traceplots for the group-level parameters for each model are shown in Figure S2 – these exhibit the "fuzzy caterpillar" shape and high degree of overlap between chains that are signatures of effective posterior sampling (Kruschke, 2014), corresponding to a dynamic but representative set of samples from the posterior. The group-level $\hat{r}$ values are all close to 1, as are the average individual-level statistics (Gelman & Rubin, 1992).

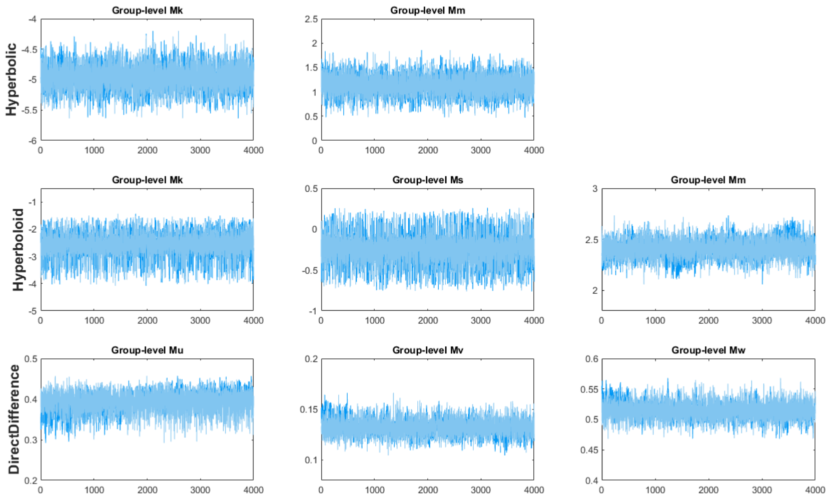

Figure S2

Trace plots for two chains of the hierarchical Bayesian MCMC approach, for each model (rows) and each parameter (columns). The x-axis of each plot indicates the step in the chain while the y-axis indicates the parameter value sampled on that step.

As we show in the main text, there are some individuals and models whose combination makes it difficult to sample robustly from the posterior. As a result, there are some $\hat{r}$ values that exceed 1.1. The group-level constraints imposed by the hierarchical model (Molloy et al., 2020),

as well as the informative priors used in the non-hierarchical Bayesian model presented in main text Figure 2, reduce but do not entirely eliminate these divergences. This is not a problem entirely unique to the Bayesian approach, as the neural networks tend to produce estimates near the mean when the parameters are difficult to estimate in the same way that the prior guides the Bayesian approaches.

Note that we also implemented the hierarchical and non-hierarchical Bayesian models using PyMC (Abril-Pla et al., 2023), to ensure that JAGS or the Gibbs sampler were not the source of misfit in the MCMC approaches. The results from PyMC are effectively identical as those produced by JAGS, indicating that the sampler is not a key issue limiting the performance of the traditional modeling approaches.

Priors from the training set

The second subject of investigation is the effect of the training set on the performance of the neural network. In practice, this training set serves a similar function to that of a Bayesian prior (Radev et al., 2020). It biases parameter estimates toward the values that are in the training set, and the model tends to lean on it more when there is less data to constrain parameter estimates.

Also like a Bayesian prior, there are several ways to minimize the influence or bias introduced by the training set. The primary way this is done is by simulating training data from a diffuse set of priors, as when using an uninformative prior in Bayesian data analyses (Kruschke, 2014). This is the approach we took in the main text, as an effort to minimize the impact of the training set of parameter estimates.

However, it is reasonable to raise concerns when there is a mismatch between the distribution of parameters in the training set and the distribution of parameters in the test set – which can happen anytime the network is applied to real data.

Typically, neural networks have more trouble with extrapolation, where they have to make predictions for outputs outside the bounds of their training set, than they do for interpolation, where they make predictions for outputs that are within or between values that they have seen in

the training set. This is a property they share with other model fitting methods as well as humans (Busemeyer et al., 2013; DeLosh et al., 1997; Barnard & Wessels, 1992). As a result, a neural network trained on a wide variety of parameter values may be more likely to succeed at fitting than a neural network trained on a relatively narrow set of parameters.

To examine how mismatch between training and test sets can impact estimation in our framework, we used two additional sets of priors to generate the parameters used to create the simulated data sets used to train the network for the direct difference model. One of these sets of priors was set with a mean that different from the true data set and a wider distribution (higher prior variance), and the other set of priors was set with a mean that differed from the true data set and a narrower distribution (lower prior variance). The exact priors were as follows. For the wide prior variance:

$$u \sim N(.5, .6) \quad (1)$$

$$v \sim N(.5, .6) \quad (2)$$

$$w \sim U(0, 1) \quad (3)$$

And for the narrow prior variance simulation:

$$u \sim N(1, .2) \quad (4)$$

$$v \sim N(1, .2) \quad (5)$$

$$w \sim U(.2, .5) \quad (6)$$

These both differed substantially from the true priors used to generate simulated data used in the main text, $u \sim N(.8, .3)$, $v \sim N(.8, .3)$, and $w \sim U(0, 1)$.

Next, we evaluated what happened if we trained neural networks on these different priors

and then applied them to a dataset generated by the opposing prior. The result is is shown in Figure S3 for four cases: where the wide prior was used to train and test the fit (vague prior, same train / test), where the narrow prior was used to both train and test the fit (specific prior, same train / test), where the wide prior was used to train the network but then tested on the data from the narrow prior (vague prior, interpolation), and where the narrow prior was used to train the network but then tested on the data from the wide prior (specific prior, extrapolation).

The latter two cases test how well the network can be expected to do when the prior or training set mismatches systematically the data to which it is ultimately applied. As shown in Figure S3, the network trained on a wide prior / training set has little difficulty across the board. Its recovery correlations are reduced only due to the reduced variance in the predictors – as a result, it effectively captures the parameters used to generate the simulated data regardless of the mismatch between training and test sets. This is because it is carrying out an *interpolation* problem – all of the parameters it fits are within the bounds of its training set.

By contrast, the neural network trained on a narrow range of simulated parameter values struggles to capture parameter values that are outside of its training set, shown in the bottom panels of Figure S3. As illustrated, it tends to predict values that are within the range of its training set, even for parameter values that were clearly outside this range. This results from problems with *extrapolation* – the network has never seen the type of data that it is asked to process, and as a result is unable to predict parameter values that are outside that same range.

Put together, these results suggest that a neural network trained on a data set with sufficiently wide prior variance should have relatively little trouble capturing the range of participant parameter values that one might realistically expect. The priors used to generate the training set should still be calibrated based on the modeler's expectations, as in all Bayesian endeavors (Kruschke, 2014; Lee, 2018), but the effect of prior mismatch is minimized when using a wide distribution of values for the training set the same as it is with vague priors.

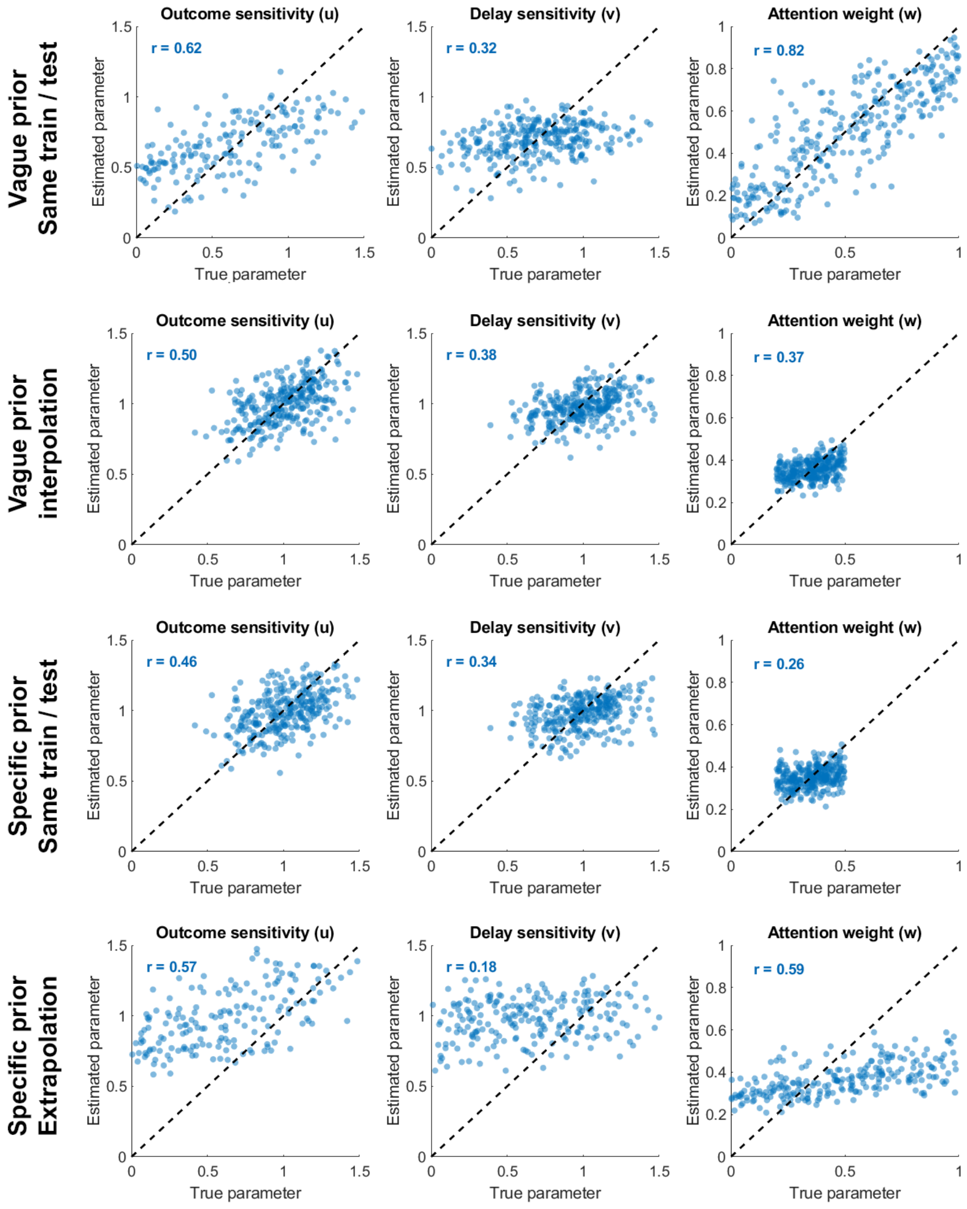

Figure S3

Effect of different priors or training sets on estimate parameters for the direct difference model. Each row corresponds to a different combination of priors used for the training set and trained network used to estimate the parameters of the model. The specific prior models were trained on a narrow set of priors, while the vague prior models were trained on a more variable set. Extrapolation corresponds to a network trained on a narrow set of priors then applied to data outside the range of its training set, while interpolation corresponds to a network trained on a wide set of priors and fit to data inside the range of its training set.

References

Abril-Pla, O., Andreani, V., Carroll, C., Dong, L., Fonnesbeck, C. J., Kochurov, M., … others (2023). Pymc: a modern, and comprehensive probabilistic programming framework in python. *PeerJ Computer Science*, *9*, e1516.

Barnard, E., & Wessels, L. (1992). Extrapolation and interpolation in neural network classifiers. *IEEE Control Systems Magazine*, *12*(5), 50–53.

Busemeyer, J. R., Byun, E., Delosh, E. L., & McDaniel, M. A. (2013). Learning functional relations based on experience with input–output pairs by humans and artificial neural networks. In *Knowledge concepts and categories* (pp. 405–437). Psychology Press.

DeLosh, E. L., Busemeyer, J. R., & McDaniel, M. A. (1997). Extrapolation: the sine qua non for abstraction in function learning. *Journal of Experimental Psychology: Learning, Memory, and Cognition*, *23*(4), 968.

Gelman, A., & Rubin, D. B. (1992). Inference from iterative simulation using multiple sequences. *Statistical Science*, *7*(4), 457–472.

Kruschke, J. K. (2014). *Doing bayesian data analysis: A tutorial with R, JAGS, and STAN.* Academic Press.

Lee, M. D. (2018). Bayesian methods in cognitive modeling. *The Stevens' Handbook of Experimental Psychology and Cognitive Neuroscience*, *5*, 37–84.

Molloy, M. F., Romeu, R. J., Kvam, P. D., Finn, P. R., Busemeyer, J., & Turner, B. M. (2020). Hierarchies improve individual assessment of temporal discounting behavior. *Decision*, *7*, 212–224.

Radev, S. T., Mertens, U. K., Voss, A., Ardizzone, L., & Köthe, U. (2020). *Bayesflow: Learning complex stochastic models with invertible neural networks.*
